# Supplementary material for: Effectiveness of targeting the health promotion settings for non-communicable disease control in low/middle-income countries: systematic review protocol
Source: BMJ Open. 2018 Jun 27;8(6):e014559. doi: 10.1136/bmjopen-2016-014559 (PMC6020988; doi:10.1136/bmjopen-2016-014559)
Supplement: Supplementary file 2 [file bmjopen-2016-014559supp002.pdf]

Supplemental File 2: **Search Strategy**

Pubmed, Embase, Ovid, Scopus databases will be searched by using specific search terms. The search terms are identified using thesaurus and other controlled vocabulary terms related to health promoting settings along with alternate terms for various diseases under NCDs and their risk factors combined with the name of the developing countries of concern. Alternate terms for developing countries like 'LMIC', 'low income country', 'lesser developed countries', 'deprived country' etc will be also be included with country names. Basically the search strategy will include four main sets which will be combined by "AND" Boolean operators. The terms within these main sets will be combined by "OR" Boolean operators.

Titles and abstracts will be searched for "Health Promoting Settings" set. All fields will be searched for "Country" set, "Disease and Risk Factor" set, "Randomised Controlled Trial" set and also for "Intervention" set.

The publication years searched will be 2000-2016.

The terms and sets of search strategy are described in table below:

Set Name	Sub-Set Terms	No. of Terms	Example
Health promoting setting Set	12 sub-sets (School, College, City, Village, Municipality or Community, Workplace, Market, Home, Hospital, Island, Prison, Old Age home)	School- 11<br>College- 23<br>City - 26<br>Village- 10<br>Municipality/ Community- 25<br>Workplace-20<br>Market-12<br>Home- 21<br>Hospital- 24<br>Island- 04<br>Prison- 17<br>Old Age home- 05<br><br>Total= 198	Primary school, govt. school, boarding school, university, educational institute, academy, metropolis, urban area, suburbs, countryside, rural area, society, locality, office, company, firm, plaza, bazaar, household, residence, clinic, nursing home, tertiary care hospital, jail, confinement place, old age home, etc.
Country Set	2 sub-sets (LMIC Country names and Alternate terms for low and middle income countries)	LMIC set - 138<br>Alternate set- 118<br><br>Total= 256	Transitional country, lower income economy, underdeveloped economy, poorer nations, deprived country, etc.
Disease & Risk Factor Set	--	59	NCDs, diabetes, stroke, cancer, CVDs, physical activity, smoking, alcohol, tobacco, diet,

			hypertension, etc.
Intervention Set	--	26	Lifestyle intervention, health behavior intervention, multiple risk factor program, etc.
Randomised Controlled Trial Set	--	16	Cluster randomized, Group randomized, Randomized,etc.
