# Supplementary material for: Effectiveness of targeting the health promotion settings for non-communicable disease control in low/middle-income countries: systematic review protocol
Source: BMJ Open. 2018 Jun 27;8(6):e014559. doi: 10.1136/bmjopen-2016-014559 (PMC6020988; doi:10.1136/bmjopen-2016-014559)
Supplement: Author's manuscript [file bmjopen-2016-014559.draft_revisions.pdf]

BMJ Open

Effectiveness of Targetting the Health Promotion Settings for NCD control in Developing Countries: Review Protocol

Journal:	*BMJ Open*
Manuscript ID	bmjopen-2016-014559
Article Type:	Protocol
Date Submitted by the Author:	03-Oct-2016
Complete List of Authors:	Jeet, Gursimer; School of Public Health, Department of Community Medicine, Post Graduate Institute of Medical Education and Research; Gursimer Jeet,<br>Thakur, JS; Post Graduate Institute of Medical Education and Research, Department of Community Medicine<br>Prinja, Shankar; Post Graduate Institute of Medical Education and Research, Community Medicine<br>Singh, Meenu; Post Graduate Institute of Medical Education and Research, Advanced Pediatric Centre<br>Kunjan, Kunjan; School of Public Health, Department of Community Medicine, Post Graduate Institute of Medical Education and Research<br>Dhadwal, Priya; Post Graduate Institute of Medical Education and Research, School of Public Health<br>Paika, Ronika; School of Public Health, Department of Community Medicine, Post Graduate Institute of Medical Education and Research
**Primary Subject Heading**:	Evidence based practice
Secondary Subject Heading:	Public health, Evidence based practice, Cardiovascular medicine, Diabetes and endocrinology
Keywords:	Targeted Interventions, NCDs, Risk Factors, Healthy settings

SCHOLARONE™
Manuscripts

Title Page

Title: Effectiveness of Targetting the Health Promotion Settings for NCD control in Developing Countries: Review Protocol

Authors: Gursimer Jeet[1]MPH, JS Thakur[1]MD, Shankar Prinja[1]MD MSc, Meenu Singh[2]MD, Kunjan Kunjan[1]BDS, Priya Dhadwal[1]BDS, Ronika Paika[1] MPH

Affiliations:

[1]School of Public Health, Post Graduate Institute of Medical Education and Research, Chandigarh, India

[2]Advanced Paediatric Centre, Post Graduate Institute of Medical Education and Research, Chandigarh, India

Type of Manuscript: Systematic Review Protocol

Word count: 242 words abstract, 3229 words main text

Keywords: Targeted Interventions, NCDs, Risk Factors, Healthy Settings,

Running Title: Targeted Interventions for Non-communicable Diseases in Health Promoting Settings

Corresponding author
Dr. Gursimer Jeet
Research Scholar,
School of Public Health,
Post Graduate Institute of Medical Education and Research, Chandigarh
Email Id: gsj_2008@yahoo.com

Contact addresses of all authors:

Dr. Gursimer Jeet
Research Scholar,
School of Public Health,
Post Graduate Institute of Medical Education and Research, Chandigarh
Email Id: gsj_2008@yahoo.com

Dr. JS Thakur
Professor of Community Medicine
School of Public Health
Post Graduate Institute of Medical Education and Research, Chandigarh
Email Id: jsthakur64@gmail.com

Dr. Shankar Prinja
Associate Professor,
School of Public Health,
Post Graduate Institute of Medical Education and Research, Chandigarh
Email Id: shankarprinja@gmail.com

For peer review only

For peer review only - http://bmjopen.bmj.com/site/about/guidelines.xhtml

Dr. Meenu Singh
Professor,
Advanced Pediatric Centre,
Post Graduate Institute of Medical Education and Research, Chandigarh
Email Id: meenusingh4@yahoo.com

Dr. Kunjan Kunjan
MPH Scholar,
School of Public Health,
Post Graduate Institute of Medical Education and Research, Chandigarh
Email Id: dr.kunjan2human@gmail.com

Dr. Priya Dhadwal
MPH Scholar,
School of Public Health,
Post Graduate Institute of Medical Education and Research, Chandigarh
Email Id: priyadhadwal.star@gmail.com

Dr. Ronika Paika
Research Scholar,
School of Public Health,
Post Graduate Institute of Medical Education and Research, Chandigarh
Email Id: ronikapaika28@gmail.com

For peer review only

ABSTRACT

Title: Effectiveness of Targetting the Health Promotion Settings for NCD control in Developing Countries: Review Protocol

INTRODUCTION:

Settings-based approaches to health promotion, involving holistic and multi-disciplinary methods which integrate action across risk factors are important. Major advantage of focusing on these settings is the continuous as well as intensive contact with the participant. Despite the apparent advantages of addressing NCDs using targeted interventions for several developed settings, a relative lack of evidence of effectiveness of such interventions in developing countries has led to poor allocation of resources towards these interventions. The focus is therefore on the setting rather than any one condition, and we therefore expect the findings to generalize to NCD prevention and control efforts. We intend to estimate the effectiveness of targeted interventions in developing countries.

METHODS AND ANALYSIS:

We will search PubMed, EMBASE, OVID, WHO Library and The Cochrane Library from the year 2000 to September, 2016 without language restrictions. Study designs to be included will be Randomised Controlled Trials. The primary outcome of effectiveness will be the percentage change in population having different behavioural risk factors. Subgroup analyses will be performed and sensitivity analyses will be conducted to assess the robustness of the findings.

ETHICS AND DISSEMINATION:

No ethical issues are foreseen. The Institute Ethics Committee of the Post Graduate Institute of Medical Education and Research approved the doctoral research protocol under which this review is being done. Dissemination will be done by submitting scientific articles to academic peer-reviewed journals. We will present the results at relevant conferences and meetings.

REGISTRATION NUMBER: PROSPERO 2016:CRD42016042647

For peer review only

For peer review only - http://bmjopen.bmj.com/site/about/guidelines.xhtml

ARTICLE SUMMARY

Strengths and limitations of the study

- The review will contribute towards evidence synthesis on effectiveness of setting based approach for reducing NCD risk factors burden in resource constrained settings.
- This will be first of its kind review synthesizing evidence from Randomized Controlled Trails on targeted settings in developing countries.
- The synthesis of evidence can probably act as an advocacy tool for better resource allocation towards settings based approach.
- Due to resource constraints focus is limited to developing countries, a more comprehensive review would have evidence available globally and comparison of developed and developing settings

For peer review only

REVIEW PROTOCOL

Effectiveness of Targetting the Health Promotion Settings for Non-communicable diseases control in Developing Countries: Review Protocol

Background

Prevalence of risk factors of non-communicable diseases (NCDs) among populations in developing countries is high.[1],[2] In 2012, NCDs contributed towards 68% of total deaths in the world with 80% of deaths in developing countries.[1] The figure is alarming than it appears as most of the NCD deaths that occurred were premature, i.e. among people aged 30-70 years.[1] In the year 2012, 48% of all deaths in South-east Asia were premature.[3] As the 4 major NCDs i.e. Cardiovascular Diseases, Diabetes, Cancer and Stroke share 4 modifiable risk factors (Tobacco, Alcohol, Diet and Physical activity), the prevention component is very crucial for this group of diseases. There is a global call to develop interventions that address the risk factors using different health promotion approaches.[4],[5]

There is good evidence that health promotion interventions focusing on changing lifestyle behaviors are more effective if conducted by targeting settings [6] rather than population based approaches alone. Therefore, settings-based approaches to health promotion, involving holistic and multi-disciplinary methods which integrates action across risk factors have become important.[4]Major advantage of focusing on these settings is the continuous as well as intensive contact with the participant. It is also proven that support from staff, suitable infrastructure and physical environment, health promoting policies have potential to positively influence the health of a person especially in these kind of settings.[7]

In order to undo the burden of globalisation and urbanisation led proximal and distal determinants of NCDs, setting based approaches may be implemented in a complete & inclusive manner by targeting singular or multiple settings simultaneously (Figure 1).These have to be guided by principles of equity, supportive environments, empowerment, community participation and multi-sectoral partnerships with strengthened health services to achieve maximum benefit in terms of disease prevention.[8] The framework given as Figure 1 helps us to understand how focusing on risk factors prevalent in the “setting” can help in finding a solution to NCDs at a larger scale. Efforts can be channelized through general population or sub-populations. Additive or multiplicative effect of working through settings can be expected, as some settings are very closely related like workplaces and homes, schools and homes. A change in behavior patterns and in turn risk profile with respect to non-communicable diseases can be expected to percolate from one setting to the other. This approach is likely to alter behavior patterns in the form of decreased risk factor levels and consequently a shift in the incidence and prevalence of cardiovascular diseases, diabetes, cancers and chronic respiratory diseases. Finally, the burden of morbidity and mortality due to non-communicable diseases can be brought to a de-escalating trend by using targeted interventions in various settings.

For peer review only - http://bmjopen.bmj.com/site/about/guidelines.xhtml

In this review, strategy of targeted interventions is being evaluated for control of NCD risk factors under different health promoting settings mentioned in the framework. The settings approach has proven effective in control of several other risk factors or diseases such as HIV[9],[10],[11] immunization[12] etc. Despite the apparent advantages of addressing NCDs using targeted interventions for several developed settings[13-16], a relative lack of evidence of effectiveness of such interventions in developing countries [17] has led to poor allocation of resources towards these interventions. The review aims to assess existing evidence on effectiveness to guide health promoting sites to accept, adopt, and correctly deliver targeted interventions for NCDs.

Methods

In reporting the protocol for this review PRISMA P checklist has been adhered to and is provided as Supplementary File along with the main manuscript (Supplementary File 1).[18] The populations of interest will be the different stakeholders involved in designing, implementation and/or sustainability of targeted NCD control interventions in different settings from developing countries. Settings here are defined as, “The place or social context in which people engage in daily activities in which environmental, organizational, and personal factors interact to affect health and wellbeing.”[19] They can be identified as having physical boundaries, a range of people with defined roles, and an organizational structure. Schools and workplaces have been popular since long as a setting for implementation of such interventions.[6] However the definition and scope of settings has widened since last few decades with acceptance of a wide range of health promoting settings, such as cities, villages, municipality/ community, workplaces, markets, home, hospitals, island, prison and old age homes.[8]

For school based targeted interventions studies set in primary, middle, high secondary, and central schools irrespective of their being private, public or aided status will be included. The inclusion criterion for mean age of students will be age between 5-18 years. For college, the trials recruiting students from institutions with, bachelor, master’s courses or other professional recognized courses will be included in the study. For schools and colleges, participants can be teaching staff and non-teaching staff, in which case trials may be classified as workplace settings. Participants may be individuals, classes, groups or whole institution.

For work site interventions, we will include trials set in government, non-government, aided institutions, organized or unorganised sectors set in developing countries. These may be involved in implementation of NCD control interventions or programs by the means of encouragement, accreditation or enforcement. Participants may be individuals, group of workers or whole institute.

Since we focus upon the settings, cluster-RCTs will be the most eligible study design for inclusion. However individual RCTs will be included if followed in any study. The controlled

For peer review only - http://bmjopen.bmj.com/site/about/guidelines.xhtml

trials, quasi experimental study designs may be the feasible design for intervention implementation, but in light of their inherent weaknesses such as lack of control area or lack of random assignment, internal validity gets reduced, and causal inference become difficult.[20] Therefore, these study designs will be excluded from the review.

Only those interventions are selected that are disseminated using setting based approach. We will include studies comparing healthy setting based strategies with target of promoting behaviours that are healthier when it comes to undertaking physical activity, patterns with respect to diet, use of tobacco, consumption of alcohol, stress, chronic disease management against usual practice or enhanced usual care. A protocol detailing methodology of this review was registered at PROSPERO.[21] Strategies of intervention may include multidisciplinary expert training groups, education and training part of strategy may involve discussions, group tasks, regular tests, etc. followed by expert group recommendations for quality improvement, reminder letters and follow up assessments for overall reduction in risk factors.[22] The interventions under scope of this process can be singular or with multiple components. These can be directed at particularly individuals, groups or complete institutions.[22 23]

Types of outcome measures

Primary outcomes

- Self- reported change in Behavioural Parameters
- Physiological or clinical parameters
- Changes in knowledge and attitudes towards NCD Risk Factors

Secondary outcomes

- Change in utilisation of health promotion related services(e.g., smoking quit-lines and counseling)
- Costs/ Cost Effectiveness of the interventions

Search methods for identifying studies: Due to the diversity of population, settings in which interventions may be implemented and outcome types, a multi-stage electronic search strategy has been developed to identify relevant publications. A draft search strategy has been submitted as Supplementary File 2. Searches of published literature on effectiveness of NCD control interventions delivered through targeted health promoting settings (focused on developing countries) will be done in the following biomedical, and general reference electronic databases, without restriction to language, however the publication year is restricted to (2000-2016): PubMed (2000–2016); Excerpta Medica Database (EMBASE) (2000–2016); OVID and World Health Organization (WHO) library. Cochrane data base of Systematic Reviews is another source of information that is proposed to be reviewed for relevant literature. World bank list

For peer review only - http://bmjopen.bmj.com/site/about/guidelines.xhtml

(2016) will be used to define the countries or regions whose data will be used for effectiveness estimation.[24]

Further related studies will be identified by examining the reference lists of articles which have been already identified through above mentioned database searches. In similar way, bibliographies of systematic and non-systematic review articles will also be examined to identify further related studies. Authors or trial investigators for further information will be contacted if any query pertaining to methodology, study outcome etc., will arise. Abstracts and full text of identified manuscripts will be reviewed. Reference lists of systematic reviews obtained in the initial scoping reviews will be screened for relevant trials. Studies examining effectiveness and those evaluating cost-effectiveness will be reviewed separately.

Searching other sources: The conference proceedings will be searched by hand where feasible (pending availability) for the last 5 years for conferences held in the field of NCDs, health promotion and health systems. In addition, trial registration Website, WHO International Clinical Trials Search Portal [25] will be searched to identify trials in process or to compare the published trials with registered protocols. In addition trial registration websites of individual developing countries such as Chinese Clinical Trials Registry[26], Clinical Trials Registry (India) [27], Clinical Research Information Service (Republic of Korea) [28], Pan African Clinical Trial Registry [29], Sri Lanka Clinical Trials Registry[30], Brazil [31]will be explored. We will also search different relevant web sources like Grey Literature Report (www.greylit.org), Eldis (www.eldis.org) for relevant grey literature.

Selection of trials and data abstraction: Two researchers (GJ/KK/PD/RP) will screen the titles and abstracts individually and in a manner independent of each other identified for relevancy. Titles and the abstracts of articles identified using searches will be imported to EndNote [32]. All of the duplicates discovered in the process will be removed. In addition to assessment of every abstract and title by both researchers, the supervisor (JS) will further independently assess 50% of abstracts. Retrieval of full text articles will be done for articles for which the selection criteria is met or declared unclear by both reviewers. If both reviewers agree to the situation of even one selection criteria not being met then full text retrieval will not be done. For the titles/abstracts where all selection criteria found categorised as ‘no’ by one reviewer were categorized entirely opposite or unclear by other reviewer, the final decision will be to retrieve the full text. Disagreements during both stages will be resolved by discussion with third reviewer (JS). Studies with a control group other than routine care and studies with a sample size <30 studies without a clearly defined intervention will be excluded. Articles written in a language other than English will be translated whenever possible. Multiple publications of the same study will be identified, grouped together and represented by a single reference.

Data extraction and management: Data will be extracted using data abstraction form which will be developed on Microsoft Office Excel 2010. The data abstraction tool will be piloted on a

For peer review only - http://bmjopen.bmj.com/site/about/guidelines.xhtml

random sample of 5 trials and modified as per the feedback from team. Data will be extracted by one reviewer (GJ) and checked against the original paper by a second independent reviewer (RP/KK/PD). Any disagreements during the process will be subject to discussion to reach a solution by discussion.

The data extraction for effectiveness assessment will be carried out in following domains: Study description (authors, aims, objectives, ethical issues); Trial details (details of location, population, intervention and control used); Intervention details (allocation methods, theoretical basis, content of intervention, delivery, duration, targeted setting); Outcome measures used and results with sufficient information; Targeted risk factors, type of setting based health promotion.

Quality as well as risk of bias assessment in included studies

Risk of bias assessment will be done by using ‘Cochrane risk of bias tool’.[33] Two authors will undertake assessment of risk of bias (GJ/PD/KK) and arbitration will be done by a third author in case of disagreements. (JS/MS/SP)

In order to assess the quality of a trial, Effective Public Health Practice Project Quality Assessment tool (EPHPP) risk of bias will be used in addition to ‘Cochrane risk of bias tool’.[34] Inter- rater reliability coefficients for exclusion as well as quality rating of the reviews will be reported.[35] The interpretation of findings will contain risk of bias assessment as risk of bias table. No restrictions will be made in the analysis on the basis of degree of risk of bias. For each primary outcome, a separate presentation of the risk of bias will be documented with respect to blinding and incomplete outcome assessment. Overall strength of evidence assessed using EPHPP tool will be presented by inclusion in 'Characteristics of included studies' table.

Inter- rater reliability coefficients for exclusion as well as quality rating of the reviews will be reported.

Data Analysis

Where data will allow, we will undertake meta-analysis. We will report the mean differences between change in intervention and control group with respect to continuous outcomes. In case measurement of same continuous outcomes has been undertaken in dissimilar manner, then we will report standardized mean differences (SMD) between the intervention and control group. If we encounter a situation where we don’t find a change per group, we will make use of end-values where randomization was successful. We will not include studies in meta-analysis in case there is non-availability of information on change per group and if there is reasonable risk of selection bias. For dichotomous outcomes, relative risk (RR) will be reported. 95% confidence intervals (CIs) will be reported with all of the effect estimates.

For peer review only - http://bmjopen.bmj.com/site/about/guidelines.xhtml

If we will be unclear whether given study is a Cluster RCT or not, the original investigator of the study will be contacted for further information. In case a situation is encountered where the original investigators have not included the clustering effect in their analyses, we will use standard methods as suggested by Cochrane group. First we will request for provision of data for individual participants, so that an intra-cluster correlation coefficient (ICC) can be calculated, and the data can be re-analyzed. In case we can't arrange for primary data, we will try to search the literature for appropriate ICC and make adjustments in the sample size in accordance with it. Correct effect estimates and standard errors from cRCTs will be pooled using generic inverse-variance method.[36] Results will be marked as re-analyzed if the data is re-analyzed. If re-analysis would not be possible, it will be mentioned clearly.

In cases where measurement of outcomes was done in participants at more than one point of time, grouping of outcomes will be undertaken for similar time points. The minimum duration for which trials must have been implemented for extraction of outcomes is 3 months.

For the interventions with multiple comparison groups, all groups that meet the inclusion criteria will be included in the review. In trials with more than two comparators, the relevant experimental and control groups will be combined to make a single pair wise comparison. If this combining the groups is not possible, we will make multiple pair wise comparisons by divide the sample size of the shared intervention group evenly across the comparison groups to avoid double counting of participants.

If case we find that data is lacking in clarity or there are missed values with respect to study methodology, participants lost to follow-up, outcome data, or statistical techniques, Primary author followed by corresponding author of the study, will be contacted through email. We will keep a condition of three reminders for requesting missing data from the authors. In case we do not a response after these, outcome will not be included for the purpose of analysis. All of the data for missing outcomes will be reported in data extraction form and in the risk of bias table.

Assessment of reporting biases

If there are more than ten trials for an outcome, the likelihood of reporting bias will be explored suing funnel plots. A visual assessment of the funnel plots will be done for sources of asymmetry such as small-study effects, publication bias, or other. If small-study effects will be found to be resulting in asymmetry, sensitivity analysis will be undertaken to show its effects on the pooled results.

Data synthesis

Detailed narrative synthesis of the results will be done for different settings and risk factors addressed. We will undertake separate meta-analyses with respect to each outcome. In order to

For peer review only - http://bmjopen.bmj.com/site/about/guidelines.xhtml

incorporate any existing heterogeneity, will use the random-effects model will be utilised for all analyses. A forest plot will be formulated for each comparison. However, the results of different outcomes under the trials will be pooled only if we find minimum of two studies and that also sufficiently homogeneous (I^2statistic < 75%). If any of the above mentioned two criterion are not met, synthesis of results will be done only in a narrative manner. We will undertake Meta-analysis using RevMan. STATA software for analyzing the data for publication bias.[37] For the primary outcomes of this review, a summary of findings table will be incorporated to take into account the number of participants and studies for each outcome, a summary of the intervention effect, and a measure of the quality of evidence for each outcome according to GRADE considerations.[38]

Subgroup analysis and investigation of heterogeneity

Outcomes of interventions identified under the review will be grouped by risk factors/diseases and type of setting. Such types of analyses will permit exploration of clinical as well as methodological heterogeneity between studies.[39] In order to make a comparison between different subgroups, we will conduct a standard heterogeneity test in RevMan 2012. This will be done by determining the I^2 statistic. Assessment of heterogeneity, or the variability among the studies included in a meta-analysis will be done using visual inspection of overlap of confidence intervals as well as by assessing statistical heterogeneity with the Chi-squared test. For I^2 statistic above 75% (an indication of substantial heterogeneity) results will not be pooled. We plan to undertake sensitivity analysis to make an assessment of how study size, study quality affect the review findings.

Discussion:

Health Promotion using Settings approach (Targeted settings) has gained importance in the past decades in developed as well as developing countries.[40] Sustained support in international health promotion research has led to generation of vast volume of literature. Through this review on targeted interventions catering a setting based approach we aim to explore their success in NCD interventions successfully and on the all four common risk factors for NCDs. The intervention delivery on NCDs in the developing countries will lead to a greater impact and different studies on different settings will help to generate greater evidence on the different targeted led interventions. These ultimately will help to conclude and generalize the targeted interventions for NCDs in different settings in developing countries. These may guide the resource allocation towards preventive and curative services using setting based approach.

For peer review only - http://bmjopen.bmj.com/site/about/guidelines.xhtml

Supplementary File 1: PRISMA P Check list

Supplementary File 2: Search Strategy

Contributorship Statement:

1. Concept and design of study: JS Thakur, Gursimer Jeet, Shankar Prinja, Meenu Singh
2. Refining the methodology and data abstraction tools: Gursimer Jeet, Kunjan Kunjan, Priya Dhadwal, Ronika Paika
3. Drafting the manuscript: Gursimer Jeet, Kunjan Kunjan, Priya Dhadwal, Ronika Paika
4. Revising manuscript critically for important intellectual content: Meenu Singh, Shankar Prinja, JS Thakur
5. Final approval of the version to be published: JS Thakur, Shankar Prinja, Meenu Singh

Competing Interests: There are no competing interests pertaining to the review.

Funding: This research is not receiving any specific grant from any funding agency in the public, commercial or not-for-profit sectors.

Data Sharing Statement: Detailed search strategy, technical appendices, statistical codes and dataset will be provided along with review and will be shared at the time of review publication.

For peer review only

For peer review only - http://bmjopen.bmj.com/site/about/guidelines.xhtml

References

1. WHO. Global Status Report on noncommunicable diseases. Geneva, Switzerland: World Health Organisation, 2014:273.
2. Thakur JS, Jeet G. Burden of Non Communicable Diseases: Mortality, Morbidity and Trends. Public Health Approaches to Non- Communicable Diseases. New Delhi: Wolters Kluwer, 2015.
3. WHO SEARO. Noncommunicable Diseases. Secondary WHO SEARO. Noncommunicable Diseases [Internet] 2016. http://www.searo.who.int/entity/noncommunicable_diseases/en/.
4. Bovet P, Paccaud. F. Cardiovascular disease and the changing face of global public health: a focus on low and middle income countries. Public Health Reviews 2012;**33**(2):397-415
5. World Health Organization. NCD Global Monitoring Framework. Geneva: World Health Organisation, 2013.
6. Verstraeten R, Roberfroid D, Lachat C, et al. Effectiveness of preventive school-based obesity interventions in low- and middle-income countries: a systematic review. The American journal of clinical nutrition 2012;**96**(2):415-38 doi: 10.3945/ajcn.112.035378[published Online First: Epub Date]|.
7. Mulligan JA, Walker D, Fox-Rushby J. Economic evaluations of non-communicable disease interventions in developing countries: a critical review of the evidence base. Cost effectiveness and resource allocation : C/E 2006;**4**:7 doi: 10.1186/1478-7547-4-7[published Online First: Epub Date]|.
8. WHO. Healthy Settings. Secondary Healthy Settings [Internet] 2016. http://www.who.int/healthy_settings/en/.
9. Clatts MC, Goldsamt LA, Giang LM, Quoc Bao L, Yu G, Colby D. Sexually transmissible infection and HIV prevention and treatment for young male sex workers in Vietnam: findings from the SHEATH intervention. Sexual health 2016 doi: 10.1071/sh16051[published Online First: Epub Date]|.
10. Wang PC, Mwango A, Moberley S, et al. A Cluster Randomised Trial on the Impact of Integrating Early Infant HIV Diagnosis with the Expanded Programme on Immunization on Immunization and HIV Testing Rates in Rural Health Facilities in Southern Zambia. PloS one 2015;**10**(10):e0141455 doi: 10.1371/journal.pone.0141455[published Online First: Epub Date]|.
11. Prinja S, Bahuguna P, Rudra S, et al. Cost effectiveness of targeted HIV prevention interventions for female sex workers in India. Sexually transmitted infections 2011;**87**(4):354-61 doi: 10.1136/sti.2010.047829[published Online First: Epub Date]|.
12. Pati S, Ladowski KL, Wong AT, Huang J, Yang J. An enriched medical home intervention using community health workers improves adherence to immunization schedules. Vaccine 2015;**33**(46):6257-63 doi: 10.1016/j.vaccine.2015.09.070[published Online First: Epub Date]|.
13. Evans CE, Albar SA, Vargas-Garcia EJ, Xu F. School-Based Interventions to Reduce Obesity Risk in Children in High- and Middle-Income Countries. Advances in food and nutrition research 2015;**76**:29-77 doi: 10.1016/bs.afnr.2015.07.003[published Online First: Epub Date]|.
14. Jago R, Rawlins E, Kipping RR, et al. Lessons learned from the AFLY5 RCT process evaluation: implications for the design of physical activity and nutrition interventions in schools. BMC public health 2015;**15**:946 doi: 10.1186/s12889-015-2293-1[published Online First: Epub Date]|.
15. Jones RA, Lubans DR, Morgan PJ, et al. School-based obesity prevention interventions: practicalities and considerations. Obesity research & clinical practice 2014;**8**(5):e497-510 doi: 10.1016/j.orcp.2013.10.004[published Online First: Epub Date]|.
16. Skouteris H, Hill B, McCabe M, Swinburn B, Busija L. A parent-based intervention to promote healthy eating and active behaviours in pre-school children: evaluation of the MEND 2-4 randomized controlled trial. Pediatric obesity 2016;**11**(1):4-10 doi: 10.1111/ijpo.12011[published Online First: Epub Date]|.

For peer review only - http://bmjopen.bmj.com/site/about/guidelines.xhtml

17. Frieden TR. A framework for public health action: the health impact pyramid. American journal of public health 2010;**100**(4):590-5 doi: 10.2105/ajph.2009.185652[published Online First: Epub Date]|.
18. Shamseer L, Moher D, Clarke M, et al. Preferred reporting items for systematic review and meta-analysis protocols (PRISMA-P) 2015: elaboration and explanation. BMJ (Clinical research ed.) 2015;**349**:g7647 doi: 10.1136/bmj.g7647[published Online First: Epub Date]|.
19. World Health Organisation. The WHO Health Promotion Glossary. Switzerland: WHO, 1998.
20. Harris AD, McGregor JC, Perencevich EN, et al. The Use and Interpretation of Quasi-Experimental Studies in Medical Informatics. Journal of the American Medical Informatics Association : JAMIA 2006;**13**(1):16-23 doi: 10.1197/jamia.M1749[published Online First: Epub Date]|.
21. Jeet G, Thakur JS, Prinja S, Singh M, Paika R. Effectiveness of targeted interventions for prevention and control of noncommunicable diseases in academic, worksite and faith based settings of developing countries. PROSPERO 2016:CRD42016042647 2016
22. Mattila R, Malmivaara A, Kastarinen M, Kivela S-L, Nissinen A. Effectiveness of multidisciplinary lifestyle intervention for hypertension: a randomised controlled trial. Journal of Human Hypertension 2003;**17**:199-205
23. Prabhakaran D, Jeemon P, Goenka S, et al. Impact of a Worksite Intervention Program on Cardiovascular Risk Factors: A Demonstration Project in an Indian Industrial Population. JACC 2009;**53**(18):1718-28
24. World Development Indicators 2016. Washington, DC: World Bank, 2016.
25. WHO. International Clinical Trials Registry Platform (ICTRP). Geneva, Switzerland.
26. Chinese Clinical Trial Register (ChiCTR). West China Hospital, Sichuan University, China: Chinese Evidence-Based Medicine Center, 2005-2015.
27. DST, WHO, ICMR. Clinical Trials Registry - India (CTRI). New Delhi: National Institute of Medical Statistics, ICMR, 2016.
28. Clinical Research Information Service (CRIS). Republic of Korea: Korea Centers for Disease Control and Prevention (KCDC), 2016.
29. SACC, EDCTP. Pan African Clinical Trials Registry (PACTR). South Africa: South African Cochrane Centre, South African Medical Research Council, 2016.
30. Sri Lanka Clinical Trials Registry. Colombo, Sri Lanka: Sri Lanka Medical Association.
31. Brazilian Ministry of Health, PAHO, FIOCRUZ. The Brazilian Clinical Trials Registry (ReBEC). Rio de Janeiro, RJ - Brazil.
32. Thomson Reuters. EndNote X6 [program]. New York, USA: Thomson Reuters, 2012.
33. Higgins JPT, Altman DG, Gøtzsche PC, et al. The Cochrane Collaboration's tool for assessing risk of bias in randomised trials. BMJ (Clinical research ed.) 2011;**343** doi: 10.1136/bmj.d5928[published Online First: Epub Date]|.
34. Armijo-Olivo S, Stiles CR, Hagen NA, Biondo PD, Cummings GG. Assessment of study quality for systematic reviews: a comparison of the Cochrane Collaboration Risk of Bias Tool and the Effective Public Health Practice Project Quality Assessment Tool: methodological research. Journal of evaluation in clinical practice 2012;**18**(1):12-8 doi: 10.1111/j.1365-2753.2010.01516.x[published Online First: Epub Date]|.
35. McHugh ML. Interrater reliability: the kappa statistic. Biochemia medica 2012;**22**(3):276-82
36. Centre for Reviews and Dissemination. Core principles and methods for conducting a systematic review of health interventions Systematic Reviews: CRD's guidance for undertaking reviews in health care: CRD, University of York, 2008.
37. StataCorp. STATA Statistical Software: Release 13 [program]. College Station, TX: STATA Corp, 2013.
38. Balshem H, Helfand M, HJ Schünemann. GRADE guidelines: Rating the quality of evidence. J Clin Epidemiol 2011;**64**:401-6

For peer review only - http://bmjopen.bmj.com/site/about/guidelines.xhtml

39. Higgins JPT, Green S. Cochrane Handbook for Systematic Reviews of Interventions. Version 5.1.0 [updated March 2011]: The Cochrane Collaboration, 2011.
40. Kokko S, Green LW, Kannas L. A review of settings-based health promotion with applications to sports clubs. Health promotion international 2014;**29**(3):494-509 doi: 10.1093/heapro/dat046[published Online First: Epub Date]|.

For peer review only

For peer review only - http://bmjopen.bmj.com/site/about/guidelines.xhtml

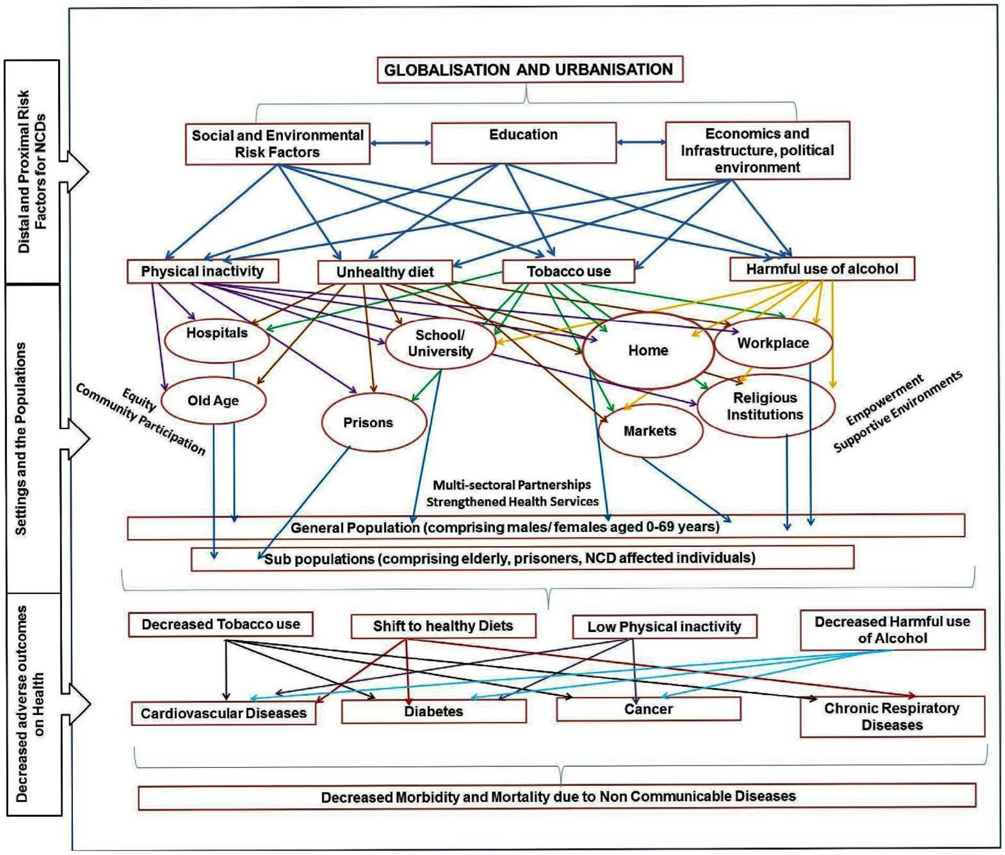

752x638mm (96 x 96 DPI)

Supplemental file 1: **Search Strategy**

Pubmed, Embase, Ovid databases will be searched by using specific search terms. The search terms are identified using thesaurus and other controlled vocabulary terms related to health promoting settings along with alternate terms for various diseases under NCDs and their risk factors combined with the name of the developing countries of concern. Alternate terms for developing countries like 'LMIC', 'low income country', 'lesser developed countries', 'deprived country' etc will be also be included with country names. Basically the search strategy will include four main sets which will be combined by "AND" Boolean operators. The terms within these main sets will be combined by "OR" Boolean operators.

Titles and abstracts will be searched for "Health Promoting Settings" set. All fields will be searched for "Country" set, "Disease and Risk Factor" set, "Randomised Controlled Trial" set and also for "Intervention" set.

The publication years searched will be 2000-2016.

The terms and sets of search strategy are described in table below:

Set Name	Sub-Set Terms	No. of Terms	Example
Health promoting setting Set	12 sub-sets (School, College, City, Village, Municipality or Community, Workplace, Market, Home, Hospital, Island, Prison, Ageing)	School- 11<br>College- 23<br>City - 26<br>Village- 10<br>Municipality/ Community- 25<br>Workplace-20<br>Market-12<br>Home- 21<br>Hospital- 24<br>Island- 04<br>Prison- 17<br>Ageing- 05<br><br>Total= 198	Primary school, govt. school, boarding school, university, educational institute, academy, metropolis, urban area, suburbs, countryside, rural area, society, locality, office, company, firm, plaza, bazaar, household, residence, clinic, nursing home, tertiary care hospital, jail, confinement place, old age home, etc.
Country Set	2 sub-sets (LMIC Country names and Alternate terms for low and middle income countries)	LMIC set - 138<br>Alternate set- 118<br><br>Total= 256	Transitional country, lower income economy, underdeveloped economy, poorer nations, deprived country, etc.
Disease & Risk Factor Set	--	59	NCDs, diabetes, stroke, cancer, CVDs, physical activity, smoking, alcohol, tobacco, diet,

For peer review only

			hypertension, etc.
Intervention Set	--	26	Lifestyle intervention, health behavior intervention, multiple risk factor program, etc.
Randomised Controlled Trial Set	--	16	Cluster randomized, Group randomized, Randomized,etc.

For peer review only

PRISMA-P (Preferred Reporting Items for Systematic review and Meta-Analysis Protocols) 2015 checklist: recommended items to address in a systematic review protocol*

Section and topic	Item No	Checklist item	Reported on Page#
**ADMINISTRATIVE INFORMATION**			
Title:			
Identification	1a	Identify the report as a protocol of a systematic review	P1 (Title)
Update	1b	If the protocol is for an update of a previous systematic review, identify as such	Not an update
Registration	2	If registered, provide the name of the registry (such as PROSPERO) and registration number	P3 (Abstract)
Authors:			
Contact	3a	Provide name, institutional affiliation, e-mail address of all protocol authors; provide physical mailing address of corresponding author	P1, P2
Contributions	3b	Describe contributions of protocol authors and identify the guarantor of the review	P12 (Contributorship Statement)
Amendments	4	If the protocol represents an amendment of a previously completed or published protocol, identify as such and list changes; otherwise, state plan for documenting important protocol amendments	NA
Support:			
Sources	5a	Indicate sources of financial or other support for the review	P12
Sponsor	5b	Provide name for the review funder and/or sponsor	NA
Role of sponsor or funder	5c	Describe roles of funder(s), sponsor(s), and/or institution(s), if any, in developing the protocol	NA
**INTRODUCTION**			
Rationale	6	Describe the rationale for the review in the context of what is already known	P6 (Background), Paragraph 1
Objectives	7	Provide an explicit statement of the question(s) the review will address with reference to participants, interventions, comparators, and outcomes (PICO)	P6, P7 (Methods)
**METHODS**			
Eligibility criteria	8	Specify the study characteristics (such as PICO, study design, setting, time frame) and report characteristics (such as years considered, language, publication status) to be used as criteria for eligibility for the review	P7, Last Paragraph
Information sources	9	Describe all intended information sources (such as electronic databases, contact with study authors, trial registers or other grey literature sources) with planned dates of coverage	P7, Last Paragraph P8, Paragraph 2

For peer review only

Search strategy	10	Present draft of search strategy to be used for at least one electronic database, including planned limits, such that it could be repeated	Supplementary File 2
Study records:			
Data management	11a	Describe the mechanism(s) that will be used to manage records and data throughout the review	P8, Paragraph 3,4<br>P9, Paragraph 1
Selection process	11b	State the process that will be used for selecting studies (such as two independent reviewers) through each phase of the review (that is, screening, eligibility and inclusion in meta-analysis)	P8, Paragraph 3,4<br>P9, Paragraph 1
Data collection process	11c	Describe planned method of extracting data from reports (such as piloting forms, done independently, in duplicate), any processes for obtaining and confirming data from investigators	P8, Paragraph 3,4<br>P10, Paragraph 4
Data items	12	List and define all variables for which data will be sought (such as PICO items, funding sources), any pre-planned data assumptions and simplifications	P9, Paragraph 1
Outcomes and prioritization	13	List and define all outcomes for which data will be sought, including prioritization of main and additional outcomes, with rationale	P7, Section: Type of Outcome Measures
Risk of bias in individual studies	14	Describe anticipated methods for assessing risk of bias of individual studies, including whether this will be done at the outcome or study level, or both; state how this information will be used in data synthesis	P9, Section: Quality as well as Risk of Bias Assessment
Data synthesis	15a	Describe criteria under which study data will be quantitatively synthesised	P10, Last Paragraph
	15b	If data are appropriate for quantitative synthesis, describe planned summary measures, methods of handling data and methods of combining data from studies, including any planned exploration of consistency (such as I^2, Kendall's τ)	P9, Section: Data Analysis<br>P10, Paragraph 1-4
	15c	Describe any proposed additional analyses (such as sensitivity or subgroup analyses, meta-regression)	P11, Paragraph 2
	15d	If quantitative synthesis is not appropriate, describe the type of summary planned	P10, Last Paragraph
Meta-bias(es)	16	Specify any planned assessment of meta-bias(es) (such as publication bias across studies, selective reporting within studies)	P10, Section: Assessment of Reporting Biases
Confidence in cumulative evidence	17	Describe how the strength of the body of evidence will be assessed (such as GRADE)	P11

***NA- Not Applicable**

From: Shamseer L, Moher D, Clarke M, Ghersi D, Liberati A, Petticrew M, Shekelle P, Stewart L, PRISMA-P Group. Preferred reporting items for systematic review and meta-analysis protocols (PRISMA-P) 2015: elaboration and explanation. BMJ. 2015 Jan 2;349(jan02 1):g7647.

For peer review only

Effectiveness of Targeting the Health Promotion Settings for Non-Communicable Disease control in Developing Countries: Systematic Review Protocol

Journal:	*BMJ Open*
Manuscript ID	bmjopen-2016-014559.R1
Article Type:	Protocol
Date Submitted by the Author:	17-Feb-2017
Complete List of Authors:	Jeet, Gursimer; School of Public Health, Department of Community Medicine, Post Graduate Institute of Medical Education and Research; Gursimer Jeet,<br>Thakur, JS; Post Graduate Institute of Medical Education and Research, Department of Community Medicine<br>Prinja, Shankar; Post Graduate Institute of Medical Education and Research, Community Medicine<br>Singh, Meenu; Post Graduate Institute of Medical Education and Research, Advanced Pediatric Centre<br>Paika, Ronika; School of Public Health, Department of Community Medicine, Post Graduate Institute of Medical Education and Research<br>Kunjan, Kunjan; School of Public Health, Department of Community Medicine, Post Graduate Institute of Medical Education and Research<br>Dhadwal, Priya; Post Graduate Institute of Medical Education and Research, School of Public Health
**Primary Subject Heading**:	Evidence based practice
Secondary Subject Heading:	Public health, Evidence based practice, Cardiovascular medicine, Diabetes and endocrinology
Keywords:	Targeted Interventions, NCDs, Risk Factors, Healthy settings

SCHOLARONE™
Manuscripts

Title Page

Title: Effectiveness of Targeting the Health Promotion Settings for Non-Communicable Disease control in Developing Countries: Systematic Review Protocol

Authors: Gursimer Jeet[1]MPH, JS Thakur[1]MD, Shankar Prinja[1]MD MSc, Meenu Singh[2]MD, Ronika Paika[1] MPH, Kunjan Kunjan[1]BDS, Priya Dhadwal[1]BDS.

Affiliations:

[1]School of Public Health, Post Graduate Institute of Medical Education and Research, Chandigarh, India

[2]Advanced Paediatric Centre, Post Graduate Institute of Medical Education and Research, Chandigarh, India

Type of Manuscript: Systematic Review Protocol

Word count: 242 words abstract, 3229 words main text

Keywords: Targeted Interventions, NCDs, Risk Factors, Healthy Settings,

Running Title: Targeted Interventions for Non-Communicable Diseases in Health Promoting Settings

Corresponding author
Dr. Gursimer Jeet
Research Scholar,
School of Public Health,
Post Graduate Institute of Medical Education and Research, Chandigarh
Email Id: gsj_2008@yahoo.com

Contact addresses of all authors:

Dr. Gursimer Jeet
Research Scholar,
School of Public Health,
Post Graduate Institute of Medical Education and Research, Chandigarh
Email Id: gsj_2008@yahoo.com

Dr. JS Thakur
Professor of Community Medicine
School of Public Health
Post Graduate Institute of Medical Education and Research, Chandigarh
Email Id: jsthakur64@gmail.com

Dr. Shankar Prinja
Associate Professor,
School of Public Health,
Post Graduate Institute of Medical Education and Research, Chandigarh
Email Id: shankarprinja@gmail.com

For peer review only

Dr. Meenu Singh
Professor,
Advanced Pediatric Centre,
Post Graduate Institute of Medical Education and Research, Chandigarh
Email Id: meenusingh4@yahoo.com

Dr. Ronika Paika
Research Scholar,
School of Public Health,
Post Graduate Institute of Medical Education and Research, Chandigarh
Email Id: ronikapaika28@gmail.com

Dr. Kunjan Kunjan
MPH Scholar,
School of Public Health,
Post Graduate Institute of Medical Education and Research, Chandigarh
Email Id: dr.kunjan2human@gmail.com

Dr. Priya Dhadwal
MPH Scholar,
School of Public Health,
Post Graduate Institute of Medical Education and Research, Chandigarh
Email Id: priyadhadwal.star@gmail.com

For peer review only

ABSTRACT

Title: Effectiveness of Targeting the Health Promotion Settings for Non-Communicable Disease control in Developing Countries: Systematic Review Protocol

INTRODUCTION:

Settings-based approaches to health promotion, involving holistic and multi-disciplinary methods which integrate action across risk factors are important. Major advantage of focusing on these settings is the continuous as well as intensive contact with the participant. Despite the apparent advantages of addressing NCDs using targeted interventions for several developed settings, a relative lack of evidence of effectiveness of such interventions in developing countries has led to poor allocation of resources towards these interventions. The focus is therefore on the settings rather than any one condition, and we therefore expect the findings to generalize to NCD prevention and control efforts. We intend to estimate the effectiveness of targeted interventions in developing countries.

METHODS AND ANALYSIS:

We will search PubMed, EMBASE, OVID, WHO Library and The Cochrane Library from the year 2000 to September, 2016 without language restrictions. Study designs to be included will be Randomised Controlled Trials. The primary outcome of effectiveness will be the percentage change in population having different behavioural risk factors. Subgroup analyses will be performed and sensitivity analyses will be conducted to assess the robustness of the findings.

ETHICS AND DISSEMINATION:

No ethical issues are foreseen. The Institute Ethics Committee of the Post Graduate Institute of Medical Education and Research approved the doctoral research protocol under which this review is being done. Dissemination will be done by submitting scientific articles to academic peer-reviewed journals. We will present the results at relevant conferences and meetings.

STUDY DESIGN: Systematic Review

REGISTRATION NUMBER: PROSPERO 2016:CRD42016042647

For peer review only

For peer review only - http://bmjopen.bmj.com/site/about/guidelines.xhtml

ARTICLE SUMMARY

Strengths and limitations of the study

- The review will contribute towards evidence synthesis on effectiveness of settings based approach for reducing NCD risk factors burden in resource constrained settings.
- This will be first of its kind review synthesizing evidence from Randomized Controlled Trails on targeted settings in developing countries.
- The synthesis of evidence can probably act as an advocacy tool for better resource allocation towards settings based approach.
- Due to resource constraints focus is limited to developing countries, a more comprehensive review would have been, synthesis of evidence available globally or comparison of the effects of the design in developed and developing settings

For peer review only

REVIEW PROTOCOL

Effectiveness of Targeting the Health Promotion Settings for Non-Communicable diseases control in Developing Countries: Review Protocol

Background

Prevalence of risk factors of non-communicable diseases (NCDs) among populations in developing countries is high.[1,2] In 2012, NCDs contributed towards 68% of total deaths in the world with 80% of deaths in developing countries.[2] The figure is alarming than it appears as most of the NCD deaths that occurred were premature, i.e. among people aged 30-70 years.[2] In the year 2012, 48% of all deaths in South-east Asia were premature.[3] As the 4 major NCDs i.e. Cardiovascular Diseases, Diabetes, Cancer and Stroke share 4 modifiable risk factors (Tobacco, Alcohol, Diet and Physical activity), the prevention component is very crucial for this group of diseases. There is a global call to develop interventions that address the risk factors using different health promotion approaches.[4,5]

There is good evidence that health promotion interventions focusing on changing lifestyle behaviors are more effective if conducted by targeting settings[6] rather than population based approaches alone. Therefore, settings-based approaches to health promotion, involving holistic and multi-disciplinary methods which integrates action across risk factors have become important.[4,7] Major advantage of focusing on these settings is the continuous as well as intensive contact with the participant. It is also proven that support from staff, suitable infrastructure and physical environment, health promoting policies have potential to positively influence the health of a person especially in these kinds of settings.[8]

In order to undo the burden of globalisation and urbanisation led proximal and distal determinants of NCDs, settings based approaches may be implemented in a complete & inclusive manner by targeting singular or multiple settings simultaneously (Figure 1).These have to be guided by principles of equity, supportive environments, empowerment, community participation and multi-sectoral partnerships with strengthened health services to achieve maximum benefit in terms of disease prevention.[9] The framework given as Figure 1 helps to understand how focusing on risk factors prevalent in the "settings" can help in finding a solution to NCDs at a larger scale. Efforts can be channelized through general population or sub-populations. Additive or multiplicative effect of working through settings can be expected, as some settings are very closely related like workplaces and homes, schools and homes. A change in behavior patterns and in turn risk profile with respect to non-communicable diseases can be expected to percolate from one setting to the other. This approach is likely to alter behavior patterns in the form of decreased risk factor levels and consequently a shift in the incidence and prevalence of cardiovascular diseases, diabetes, cancers and chronic respiratory diseases. Finally, the rate of increase in burden of morbidity and mortality due to non-communicable diseases can slowed down by using targeted interventions in various settings.

For peer review only - http://bmjopen.bmj.com/site/about/guidelines.xhtml

In this review, strategy of targeted interventions is being evaluated for control of NCD risk factors under different health promoting settings mentioned in the framework. The settings approach has proven effective in control of several other risk factors or diseases such as HIV[10-12], immunization[13] etc. Despite the apparent advantages of addressing NCDs using targeted interventions for several developed settings[14-17], a relative lack of evidence of effectiveness of such interventions in developing countries[18] has led to poor allocation of resources towards these interventions. The systematic review aims to assess existing evidence on effectiveness to guide health promoting sites to accept, adopt, and correctly deliver targeted interventions for NCDs.

Methods

In reporting the protocol for this review PRISMA P checklist has been adhered to and is provided as a Supplementary File along with the main manuscript (Supplementary File 1).[19]

Population: The populations of interest will be the different stakeholders involved in design, implementation and sustainability of targeted NCD control interventions in different settings from developing countries.

Intervention details: Interventions here refer to use of "settings based approach for delivery of health promotion interventions." Settings here are defined as, "The place or social context in which people engage in daily activities in which environmental, organizational, and personal factors interact to affect health and wellbeing."[20] They can be identified as having physical boundaries, a range of people with defined roles, and an organizational structure. Schools and workplaces have been popular since long as a setting for implementation of such interventions.[6] However the definition and scope of settings has widened since last few decades with acceptance of a wide range of health promoting settings, such as cities, villages, municipality/ community, workplaces, markets, home, hospitals, islands, prisons and old age homes.[9]

For school based targeted interventions studies in primary, middle, high secondary, and central schools (a selective secondary education school in some countries or the schools that are affiliated to central government in some countries) irrespective of their being private, public or aided (school receiving a part of its maintenance costs from public funds but retaining control over appointments and religious instruction) status will be included. The inclusion criterion for mean age of students will be age between 5-18 years. For college, the trials recruiting students from institutions with bachelor, master's courses or other professional recognized courses will be included in the study. For schools and colleges, participants can be teaching staff and non-teaching staff, in which case trials may be classified as workplace settings. Participants may be individuals, classes, groups or whole institution.

For work site interventions, we will include trials set in government, non-government, aided institutions, organized or unorganised sectors set in developing countries. These may be involved

For peer review only - http://bmjopen.bmj.com/site/about/guidelines.xhtml

in implementation of NCD control interventions or programs by the means of encouragement, accreditation or enforcement. Participants may be individuals, group of workers or whole institute.

Comparator: Routine care or enhanced routine care

Inclusion and Exclusion Criterion: Since we focus upon the settings, cluster-RCTs will be the most eligible study design for inclusion. However individual RCTs will be included if followed in any study. The controlled trials, quasi experimental study designs may be the feasible design for intervention implementation, but in light of their inherent weaknesses such as lack of control area or lack of random assignment, internal validity gets reduced, and causal inference becomes difficult.[21] Therefore, these study designs will be excluded from the review.

Only those interventions are selected that are disseminated using settings based approach. We will include studies comparing healthy settings based strategies with target of promoting behaviours that are healthier when it comes to undertaking physical activity, patterns with respect to diet, use of tobacco, consumption of alcohol against usual practice or enhanced usual care. A protocol detailing methodology of this review was registered at PROSPERO.[22] Strategies of intervention may include multidisciplinary expert training groups, education and training part of strategy may involve discussions, group tasks, regular tests, etc. followed by expert group recommendations for quality improvement, reminder letters and follow up assessments for overall reduction in risk factors.[23] The interventions under scope of this process can be singular or with multiple components. These can be directed at particularly individuals, groups or complete institutions.[23 ,24] The model of delivery of interventions can belong to (but not limited to) change in organisation policies or practices, enhanced family support, social networking, change in physical environment or routine practices.

Types of outcome measures: Selection of outcomes for the review is guided by nature of interventions under study which focus primarily on promoting the healthy behaviours. We have selected change in participant behaviour and a consequent shift in their physical (e.g. weight, blood pressure), biochemical or cognitive parameters as primary outcomes as these are directly related to participant’s health status. Additionally, as secondary outcomes we will report rate of use of services or cost/ cost effectiveness of implementing interventions

Primary outcomes

- Self- reported change in Behavioural Parameters
- Changes in Physiological or clinical parameters
- Changes in knowledge and attitudes towards NCD Risk Factors

For peer review only

For peer review only - http://bmjopen.bmj.com/site/about/guidelines.xhtml

Secondary outcomes (outcomes reported along with health outcomes)

- Change in utilisation of health promotion related services(e.g., smoking quit-lines and counseling)
- Costs/ Cost Effectiveness of the interventions

Search methods for identifying studies: Due to the diversity of population, settings in which interventions may be implemented and outcome types, a multi-stage electronic search strategy has been developed to identify relevant publications. A draft search strategy has been submitted as Supplementary File 2. Searches of published literature on effectiveness of NCD control interventions delivered through targeted health promoting settings (focused on developing countries) will be done in the following biomedical, and general reference electronic databases, without restriction to language, however the publication year is restricted to (2000-2016): PubMed (2000–2016); Excerpta Medica Database (EMBASE) (2000–2016); OVID and World Health Organization (WHO) library. Cochrane data base of Systematic Reviews is another source of information that is proposed to be reviewed for relevant literature. The time period has been chosen considering the evidence that NCD prevention and control priorities have been set recently in developing countries primarily after World Health Organization's call for global action. World Bank list (2016) will be used to define the countries or regions whose data will be used for effectiveness estimation.[25]

Further related studies will be identified by examining the reference lists of articles which have been already identified through above mentioned database searches. In similar way, bibliographies of systematic and non-systematic review articles will also be examined to identify further related studies. Authors or trial investigators for further information will be contacted if any query pertaining to methodology, study outcome etc., will arise. Abstracts and full text of identified manuscripts will be reviewed. Reference lists of systematic reviews obtained in the initial scoping reviews will be screened for relevant trials. Studies examining effectiveness and those evaluating cost-effectiveness will be reviewed separately.

Searching other sources: The conference proceedings will be searched by hand where ever feasible (pending availability) for the last 5 years for conferences held in the field of NCDs, health promotion and health systems. In addition, trial registration Website, WHO International Clinical Trials Search Portal[26] will be searched to identify trials in process or to compare the published trials with registered protocols. In addition trial registration websites of individual developing countries such as Chinese Clinical Trials Registry[27], Clinical Trials Registry (India)[28], Clinical Research Information Service (Republic of Korea)[29], Pan African Clinical Trial Registry[30], Sri Lanka Clinical Trials Registry[31], Brazil[32] will be explored. We will also

For peer review only - http://bmjopen.bmj.com/site/about/guidelines.xhtml

search different relevant web sources like Grey Literature Report (www.greylit.org), Eldis (www.eldis.org) for relevant grey literature.

Selection of trials and data abstraction: Two researchers (GJ/KK/PD/RP) will screen the titles and abstracts individually and in a manner independent of each other identified for relevancy. Titles and the abstracts of articles identified using searches will be imported to EndNote.[33] All of the duplicates discovered in the process will be removed. In addition to assessment of every abstract and title by both researchers, the supervisor (JS) will further independently assess 50% of abstracts. Retrieval of full text articles will be done for articles for which the selection criteria is met or declared unclear by both reviewers. If both reviewers agree to the situation of even one selection criteria not being met then full text retrieval will not be done. For the titles/abstracts where all selection criteria found categorised as 'no' by one reviewer were categorized entirely opposite or unclear by other reviewer, the final decision will be to retrieve the full text of the article for the better review of selection criteria and if any disagreements found during both stages then that will be resolved by discussion with third reviewer (JS). Studies with a control group other than routine care, studies with a sample size <30 and studies without a clearly defined intervention will be excluded. Articles written in a language other than English will be translated whenever possible. Multiple publications of the same study will be identified, grouped together and represented by a single reference.

Data extraction and management: Data will be extracted using data abstraction form which will be developed on Microsoft Office Excel 2010. The data abstraction tool will be piloted on a random sample of 5 trials and modified as per the feedback from team. Data will be extracted by one reviewer (GJ) and checked against the original paper by a second independent reviewer (RP/KK/PD). Any disagreements during the process will be subject to discussion to reach a solution by discussion and if not resolved by discussion between the two then the issue will be resolved as per final decision of the third reviewer (JS).

The data extraction for effectiveness assessment will be carried out in following domains: Study description (authors, aims, objectives, ethical issues); Trial details (details of location, population, intervention and control used); Intervention details (allocation methods, theoretical basis, content of intervention, delivery, duration, targeted setting); Outcome measures used and results with sufficient information; Targeted risk factors, type of settings based health promotion.

Quality as well as risk of bias assessment in included studies

Risk of bias assessment will be done by using 'Cochrane risk of bias tool'.[34] Two authors will undertake assessment of risk of bias (GJ/PD/KK) and arbitration will be done by a third author in case of disagreements. (JS/MS/SP)

For peer review only - http://bmjopen.bmj.com/site/about/guidelines.xhtml

In order to assess the quality of a trial, Effective Public Health Practice Project Quality Assessment tool (EPHPP) risk of bias tool will be used in addition to 'Cochrane risk of bias tool'.[35] Inter- rater reliability coefficients for exclusion as well as quality rating of the reviews will be reported.[36] The interpretation of findings will contain risk of bias assessment as risk of bias table. No restrictions will be made in the analysis on the basis of degree of risk of bias. For each primary outcome, a separate presentation of the risk of bias will be documented with respect to blinding and incomplete outcome assessment. Overall strength of evidence assessed using EPHPP tool will be presented by inclusion in 'Characteristics of included studies' table.

Data Analysis

Where data will allow, we will undertake meta-analysis. We will report the mean differences between change in intervention and control group with respect to continuous outcomes. In case measurement of same continuous outcomes has been undertaken in dissimilar manner, then we will report standardized mean differences (SMD) between the intervention and control group. If we encounter a situation where we don't find a change per group, we will make use of end-values where randomization was successful. We will not include studies in meta-analysis in case there is non-availability of information on change per group and if there is reasonable risk of selection bias. For dichotomous outcomes, relative risk (RR) will be reported. 95% confidence intervals (CIs) will be reported with all of the effect estimates.

If it is unclear whether given study is a Cluster RCT or not, the original investigator of the study will be contacted for further information. In case a situation is encountered where the original investigators have not included the clustering effect in their analyses, we will use standard methods as suggested by Cochrane group. First we will request for provision of data for individual participants, so that an intra-cluster correlation coefficient (ICC) can be calculated, and the data can be re-analyzed. In case we can't get primary data, we will try to search the literature for appropriate ICC and make adjustments in the sample size in accordance with it. Correct effect estimates and standard errors from cRCTs(Cluster randomized controlled trials) will be pooled using generic inverse-variance method.[37] Results will be marked as re-analyzed if the data is re-analyzed. If re-analysis would not be possible, it will be mentioned clearly.

In cases where measurement of outcomes was done in participants at more than one point of time, grouping of outcomes will be undertaken for similar time points. The minimum duration for which trials must have been implemented for extraction of outcomes is 3 months.

For the interventions with multiple comparison groups, all groups that meet the inclusion criteria will be included in the review. In trials with more than two comparisons , the relevant experimental and control groups will be combined to make a single pair wise comparison. If this combining of the groups is not possible, we will make multiple pair wise comparisons by

For peer review only - http://bmjopen.bmj.com/site/about/guidelines.xhtml

dividing the sample size of the shared intervention group evenly across the comparison groups to avoid double counting of participants.

If case we find that data is lacking in clarity or there are missed values with respect to study methodology, participants lost to follow-up, outcome data, or statistical techniques, primary author followed by corresponding author of the study, will be contacted through email. We will keep a condition of three reminders for requesting missing data from the authors. In case we do not obtain a response after these, outcome will not be included for the purpose of analysis. All of the data for missing outcomes will be reported in data extraction form and in the risk of bias table.

Assessment of reporting biases

If there are more than ten trials for an outcome, the likelihood of reporting bias will be explored by using funnel plots. A visual assessment of the funnel plots will be done for sources of asymmetry such as small-study effects, publication bias, or other. If small-study effects will found to be resulting in asymmetry then further sensitivity analysis will be undertaken to show its effects on the pooled results.

Data synthesis

Detailed narrative synthesis of the results will be done for different settings and risk factors addressed. We will undertake separate meta-analyses with respect to each outcome. In order to incorporate any existing heterogeneity, the random-effects model will be utilized. A forest plot will be formulated for each comparison. However, the results of different outcomes under the trials will be pooled only if we find minimum of two studies and the studies are sufficiently homogeneous (I^2statistic < 75%). If either of the above mentioned two criterion are not met, synthesis of results will be done only in a narrative manner. We will undertake Meta-analysis using RevMan. STATA software for analyzing the data for publication bias because the publication bias tests as Eggers test are not applicable in RevMan.[38] For the primary outcomes of this review, a summary of findings table will be incorporated to take into account the number of participants and studies for each outcome, a summary of the intervention effect, and a measure of the quality of evidence for each outcome according to GRADE considerations.[39]

Subgroup analysis and investigation of heterogeneity

Outcomes of interventions identified under the review will be grouped by risk factors/diseases and type of setting. Such types of analyses will permit exploration of clinical as well as methodological heterogeneity between studies.[40] In order to make a comparison between different subgroups, we will conduct a standard heterogeneity test in RevMan 2012. This will be done by determining the I^2 statistic. Assessment of heterogeneity, or the variability among the

For peer review only - http://bmjopen.bmj.com/site/about/guidelines.xhtml

studies included in a meta-analysis will be done using visual inspection of overlap of confidence intervals as well as by assessing statistical heterogeneity with the Chi-squared test. For I^2 statistic above 75% (an indication of substantial heterogeneity) results will not be pooled. We plan to undertake sensitivity analysis to make an assessment of how study size, study quality affect the review findings.

Ethics and dissemination:

No ethical issues are foreseen. The Institute Ethics Committee of the Post Graduate Institute of Medical Education and Research approved the doctoral research protocol under which this review is being done. Dissemination will be done by submitting scientific articles to academic peer-reviewed journals. We will present the results at relevant conferences and meetings.

Discussion:

Sustained support in international health promotion research has led to generation of vast volume of literature on settings based approach. Through this review we aim to explore their success in NCD prevention. This will help to conclude and generalize the targeted interventions for NCDs in different settings among developing countries. Thus, ultimately guiding the resource allocation towards preventive and curative services using settings based approach.

For peer review only

For peer review only - http://bmjopen.bmj.com/site/about/guidelines.xhtml

Supplementary File 1: PRISMA P Check list

Supplementary File 2: Search Strategy

Contributorship Statement:

1. Concept and design of study: JS Thakur, Gursimer Jeet, Shankar Prinja, Meenu Singh
2. Refining the methodology and data abstraction tools: Gursimer Jeet, Kunjan Kunjan, Priya Dhadwal, Ronika Paika
3. Drafting the manuscript: Gursimer Jeet, Kunjan Kunjan, Priya Dhadwal, Ronika Paika
4. Revising manuscript critically for important intellectual content: Meenu Singh, Shankar Prinja, JS Thakur
5. Final approval of the version to be published: JS Thakur, Shankar Prinja, Meenu Singh

Competing Interests: There are no competing interests pertaining to the review.

Funding: This research is not receiving any specific grant from any funding agency in the public, commercial or not-for-profit sectors.

Data Sharing Statement: Detailed search strategy, technical appendices, statistical codes and dataset will be provided along with review and will be shared at the time of review publication.

For peer review only

References

1. Thakur JS, Jeet G. Burden of Non Communicable Diseases: Mortality, Morbidity and Trends. Public Health Approaches to Non- Communicable Diseases. New Delhi: Wolters Kluwer 2015.
2. WHO. Global Status Report on noncommunicable diseases. Geneva, Switzerland: World Health Organisation, 2014:273.
3. WHO SEARO. Noncommunicable Diseases [Internet]. New Delhi: WHO SEARO; 2016 [cited 2016 September 26]. Available from: http://www.searo.who.int/entity/noncommunicable_diseases/en/28/01/2017.
4. Bovet P, Paccaud. F. Cardiovascular disease and the changing face of global public health: a focus on low and middle income countries. *Public Health Reviews* 2012;33(2):397-415.
5. World Health Organization. NCD Global Monitoring Framework. [Internet]. Geneva: World Health Organisation; 2013 [28/01/2017]. Available from: http://www.who.int/nmh/global_monitoring_framework/en/27/01/2017.
6. Verstraeten R, Roberfroid D, Lachat C, et al. Effectiveness of preventive school-based obesity interventions in low- and middle-income countries: a systematic review. *The American journal of clinical nutrition* 2012;96(2):415-38. doi: 10.3945/ajcn.112.035378 [published Online First: 2012/07/05]
7. Kokko S, Green LW, Kannas L. A review of settings-based health promotion with applications to sports clubs. *Health promotion international* 2014;29(3):494-509. doi: 10.1093/heapro/dat046 [published Online First: 2013/07/03]
8. Mulligan JA, Walker D, Fox-Rushby J. Economic evaluations of non-communicable disease interventions in developing countries: a critical review of the evidence base. *Cost effectiveness and resource allocation : C/E* 2006;4:7. doi: 10.1186/1478-7547-4-7 [published Online First: 2006/04/06]
9. WHO. Healthy Settings [Internet]. Geneva2016 [Available from: http://www.who.int/healthy_settings/en/ accessed 23/09/2016.
10. Clatts MC, Goldsamt LA, Giang LM, et al. Sexually transmissible infection and HIV prevention and treatment for young male sex workers in Vietnam: findings from the SHEATH intervention. *Sexual health* 2016 doi: 10.1071/sh16051 [published Online First: 2016/09/09]
11. Wang PC, Mwango A, Moberley S, et al. A Cluster Randomised Trial on the Impact of Integrating Early Infant HIV Diagnosis with the Expanded Programme on Immunization on Immunization and HIV Testing Rates in Rural Health Facilities in Southern Zambia. *PloS one* 2015;10(10):e0141455. doi: 10.1371/journal.pone.0141455 [published Online First: 2015/10/30]
12. Prinja S, Bahuguna P, Rudra S, et al. Cost effectiveness of targeted HIV prevention interventions for female sex workers in India. *Sexually transmitted infections* 2011;87(4):354-61. doi: 10.1136/sti.2010.047829 [published Online First: 2011/03/31]
13. Pati S, Ladowski KL, Wong AT, et al. An enriched medical home intervention using community health workers improves adherence to immunization schedules. *Vaccine* 2015;33(46):6257-63. doi: 10.1016/j.vaccine.2015.09.070 [published Online First: 2015/10/06]

For peer review only - http://bmjopen.bmj.com/site/about/guidelines.xhtml

14. Evans CE, Albar SA, Vargas-Garcia EJ, et al. School-Based Interventions to Reduce Obesity Risk in Children in High- and Middle-Income Countries. *Advances in food and nutrition research* 2015;76:29-77. doi: 10.1016/bs.afnr.2015.07.003 [published Online First: 2015/11/26]
15. Jago R, Rawlins E, Kipping RR, et al. Lessons learned from the AFLY5 RCT process evaluation: implications for the design of physical activity and nutrition interventions in schools. *BMC public health* 2015;15:946. doi: 10.1186/s12889-015-2293-1 [published Online First: 2015/09/25]
16. Jones RA, Lubans DR, Morgan PJ, et al. School-based obesity prevention interventions: practicalities and considerations. *Obesity research & clinical practice* 2014;8(5):e497-510. doi: 10.1016/j.orcp.2013.10.004 [published Online First: 2014/09/30]
17. Skouteris H, Hill B, McCabe M, et al. A parent-based intervention to promote healthy eating and active behaviours in pre-school children: evaluation of the MEND 2-4 randomized controlled trial. *Pediatric obesity* 2016;11(1):4-10. doi: 10.1111/ijpo.12011 [published Online First: 2015/02/28]
18. Frieden TR. A framework for public health action: the health impact pyramid. *American journal of public health* 2010;100(4):590-5. doi: 10.2105/ajph.2009.185652 [published Online First: 2010/02/20]
19. Shamseer L, Moher D, Clarke M, et al. Preferred reporting items for systematic review and meta-analysis protocols (PRISMA-P) 2015: elaboration and explanation. *BMJ (Clinical research ed)* 2015;349:g7647. doi: 10.1136/bmj.g7647 [published Online First: 2015/01/04]
20. World Health Organisation. The WHO Health Promotion Glossary. Switzerland: WHO, 1998.
21. Harris AD, McGregor JC, Perencevich EN, et al. The Use and Interpretation of Quasi-Experimental Studies in Medical Informatics. *Journal of the American Medical Informatics Association : JAMIA* 2006;13(1):16-23. doi: 10.1197/jamia.M1749
22. Jeet G, Thakur JS, Prinja S, et al. Effectiveness of targeted interventions for prevention and control of noncommunicable diseases in academic, worksite and faith based settings of developing countries. *PROSPERO 2016:CRD42016042647* 2016
23. Mattila R, Malmivaara A, Kastarinen M, et al. Effectiveness of multidisciplinary lifestyle intervention for hypertension: a randomised controlled trial. *Journal of Human Hypertension* 2003;17:199-205.
24. Prabhakaran D, Jeemon P, Goenka S, et al. Impact of a Worksite Intervention Program on Cardiovascular Risk Factors: A Demonstration Project in an Indian Industrial Population. *JACC* 2009;53(18):1718-28.
25. The World Bank. World Bank Country and Lending Groups [Internet]. Washington, DC: World Bank; 2016 [Available from: https://datahelpdesk.worldbank.org/knowledgebase/articles/906519-world-bank-country-and-lending-groups27/01/2017.
26. WHO. International Clinical Trials Registry Platform (ICTRP). Geneva, Switzerland.
27. Chinese Clinical Trial Register (ChiCTR). West China Hospital, Sichuan University, China: Chinese Evidence-Based Medicine Center, 2005-2015.
28. DST, WHO, ICMR. Clinical Trials Registry - India (CTRI). New Delhi: National Institute of Medical Statistics, ICMR, 2016.

For peer review only - http://bmjopen.bmj.com/site/about/guidelines.xhtml

29. Clinical Research Information Service (CRIS). Republic of Korea: Korea Centers for Disease Control and Prevention (KCDC), 2016.
30. SACC, EDCTP. Pan African Clinical Trials Registry (PACTR). South Africa: South African Cochrane Centre, South African Medical Research Council, 2016.
31. Sri Lanka Clinical Trials Registry. Colombo, Sri Lanka: Sri Lanka Medical Association.
32. Brazilian Ministry of Health, PAHO, FIOCRUZ. The Brazilian Clinical Trials Registry (ReBEC). Rio de Janeiro, RJ - Brazil.
33. Thomson Reuters. EndNote X6 [program]. New York, USA: Thomson Reuters, 2012.
34. Higgins JPT, Altman DG, Gøtzsche PC, et al. The Cochrane Collaboration's tool for assessing risk of bias in randomised trials. *BMJ (Clinical research ed)* 2011;343 doi: 10.1136/bmj.d5928
35. Armijo-Olivo S, Stiles CR, Hagen NA, et al. Assessment of study quality for systematic reviews: a comparison of the Cochrane Collaboration Risk of Bias Tool and the Effective Public Health Practice Project Quality Assessment Tool: methodological research. *Journal of evaluation in clinical practice* 2012;18(1):12-8. doi: 10.1111/j.1365-2753.2010.01516.x [published Online First: 2010/08/12]
36. McHugh ML. Interrater reliability: the kappa statistic. *Biochemia medica* 2012;22(3):276-82. [published Online First: 2012/10/25]
37. Centre for Reviews and Dissemination. Core principles and methods for conducting a systematic review of health interventions Systematic Reviews: CRD's guidance for undertaking reviews in health care: CRD, University of York 2008.
38. StataCorp. STATA Statistical Software: Release 13 [program]. College Station, TX: STATA Corp, 2013.
39. Balshem H, Helfand M, HJ Schünemann. GRADE guidelines: Rating the quality of evidence. *J Clin Epidemiol* 2011;64:401-6.
40. Higgins JPT, Green S. Cochrane Handbook for Systematic Reviews of Interventions. Version 5.1.0 [updated March 2011]: The Cochrane Collaboration, 2011.

For peer review only

For peer review only - http://bmjopen.bmj.com/site/about/guidelines.xhtml

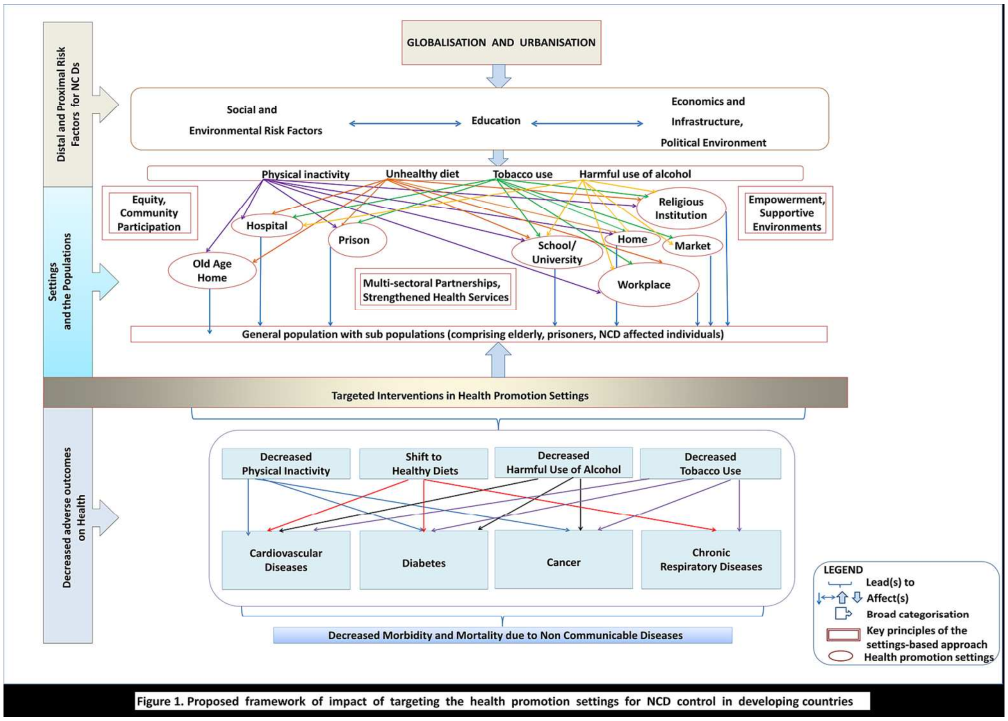

Proposed framework of impact of targeting the health promotion settings for NCD control in developing countries

155x110mm (300 x 300 DPI)

Supplemental File 2: **Search Strategy**

Pubmed, Embase, Ovid, Scopus databases will be searched by using specific search terms. The search terms are identified using thesaurus and other controlled vocabulary terms related to health promoting settings along with alternate terms for various diseases under NCDs and their risk factors combined with the name of the developing countries of concern. Alternate terms for developing countries like 'LMIC', 'low income country', 'lesser developed countries', 'deprived country' etc will be also be included with country names. Basically the search strategy will include four main sets which will be combined by "AND" Boolean operators. The terms within these main sets will be combined by "OR" Boolean operators.

Titles and abstracts will be searched for "Health Promoting Settings" set. All fields will be searched for "Country" set, "Disease and Risk Factor" set, "Randomised Controlled Trial" set and also for "Intervention" set.

The publication years searched will be 2000-2016.

The terms and sets of search strategy are described in table below:

Set Name	Sub-Set Terms	No. of Terms	Example
Health promoting setting Set	12 sub-sets (School, College, City, Village, Municipality or Community, Workplace, Market, Home, Hospital, Island, Prison, Old Age home)	School- 11<br>College- 23<br>City - 26<br>Village- 10<br>Municipality/ Community- 25<br>Workplace-20<br>Market-12<br>Home- 21<br>Hospital- 24<br>Island- 04<br>Prison- 17<br>Old Age home- 05<br><br>Total= 198	Primary school, govt. school, boarding school, university, educational institute, academy, metropolis, urban area, suburbs, countryside, rural area, society, locality, office, company, firm, plaza, bazaar, household, residence, clinic, nursing home, tertiary care hospital, jail, confinement place, old age home, etc.
Country Set	2 sub-sets (LMIC Country names and Alternate terms for low and middle income countries)	LMIC set - 138<br>Alternate set- 118<br><br>Total= 256	Transitional country, lower income economy, underdeveloped economy, poorer nations, deprived country, etc.
Disease & Risk Factor Set	--	59	NCDs, diabetes, stroke, cancer, CVDs, physical activity, smoking, alcohol, tobacco, diet,

For peer review only

			hypertension, etc.
Intervention Set	--	26	Lifestyle intervention, health behavior intervention, multiple risk factor program, etc.
Randomised Controlled Trial Set	--	16	Cluster randomized, Group randomized, Randomized,etc.

For peer review only

Supplementary File 1: PRISMA-P (Preferred Reporting Items for Systematic review and Meta-Analysis Protocols) 2015 checklist: recommended items to address in a systematic review protocol*

Section and topic	Item No	Checklist item	Reported on Page#
**ADMINISTRATIVE INFORMATION**			
Title:			
Identification	1a	Identify the report as a protocol of a systematic review	P1 (Title)
Update	1b	If the protocol is for an update of a previous systematic review, identify as such	Not an update
Registration	2	If registered, provide the name of the registry (such as PROSPERO) and registration number	P3 (Abstract)
Authors:			
Contact	3a	Provide name, institutional affiliation, e-mail address of all protocol authors; provide physical mailing address of corresponding author	P1, P2
Contributions	3b	Describe contributions of protocol authors and identify the guarantor of the review	P13(Contributorship Statement)
Amendments	4	If the protocol represents an amendment of a previously completed or published protocol, identify as such and list changes; otherwise, state plan for documenting important protocol amendments	NA
Support:			
Sources	5a	Indicate sources of financial or other support for the review	P13 (Funding)
Sponsor	5b	Provide name for the review funder and/or sponsor	NA
Role of sponsor or funder	5c	Describe roles of funder(s), sponsor(s), and/or institution(s), if any, in developing the protocol	NA
**INTRODUCTION**			
Rationale	6	Describe the rationale for the review in the context of what is already known	P6 (Background), Paragraph 1
Objectives	7	Provide an explicit statement of the question(s) the review will address with reference to participants, interventions, comparators, and outcomes (PICO)	P6, P7 (Methods)
**METHODS**			
Eligibility criteria	8	Specify the study characteristics (such as PICO, study design, setting, time frame) and report characteristics (such as years considered, language, publication status) to be used as criteria for eligibility for the review	P7, Last Paragraph
Information sources	9	Describe all intended information sources (such as electronic databases, contact with study authors, trial registers or other grey literature sources) with planned dates of coverage	P8
Search strategy	10	Present draft of search strategy to be used for at least one electronic database, including planned limits, such that it	Supplementary File

For peer review only

		could be repeated	2
Study records:			
Data management	11a	Describe the mechanism(s) that will be used to manage records and data throughout the review	P8 last lines, P9 Paragraph 1 (Data Extraction and Management)
Selection process	11b	State the process that will be used for selecting studies (such as two independent reviewers) through each phase of the review (that is, screening, eligibility and inclusion in meta-analysis)	P9
Data collection process	11c	Describe planned method of extracting data from reports (such as piloting forms, done independently, in duplicate), any processes for obtaining and confirming data from investigators	P8
Data items	12	List and define all variables for which data will be sought (such as PICO items, funding sources), any pre-planned data assumptions and simplifications	P9, Line 268-272
Outcomes and prioritization	13	List and define all outcomes for which data will be sought, including prioritization of main and additional outcomes, with rationale	P7 Line 205, P8 Line 210-212
Risk of bias in individual studies	14	Describe anticipated methods for assessing risk of bias of individual studies, including whether this will be done at the outcome or study level, or both; state how this information will be used in data synthesis	P9, Section: Quality as well as Risk of Bias Assessment
Data synthesis	15a	Describe criteria under which study data will be quantitatively synthesised	P10
	15b	If data are appropriate for quantitative synthesis, describe planned summary measures, methods of handling data and methods of combining data from studies, including any planned exploration of consistency (such as I^2, Kendall's τ)	P11
	15c	Describe any proposed additional analyses (such as sensitivity or subgroup analyses, meta-regression)	P11
	15d	If quantitative synthesis is not appropriate, describe the type of summary planned	P11 Line 327, 336-338
Meta-bias(es)	16	Specify any planned assessment of meta-bias(es) (such as publication bias across studies, selective reporting within studies)	P11, Line 321-325
Confidence in cumulative evidence	17	Describe how the strength of the body of evidence will be assessed (such as GRADE)	P11, Line 338

***NA- Not Applicable**

From: Shamseer L, Moher D, Clarke M, Ghersi D, Liberati A, Petticrew M, Shekelle P, Stewart L, PRISMA-P Group. Preferred reporting items for systematic review and meta-analysis protocols (PRISMA-P) 2015: elaboration and explanation. BMJ. 2015 Jan 2;349(jan02 1):g7647.

For peer review only

BMJ Open

Effectiveness of Targeting the Health Promotion Settings for Non-Communicable Disease Control in Developing Countries: Systematic Review Protocol

Journal:	*BMJ Open*
Manuscript ID	bmjopen-2016-014559.R2
Article Type:	Protocol
Date Submitted by the Author:	15-Apr-2017
Complete List of Authors:	Jeet, Gursimer; School of Public Health, Department of Community Medicine, Post Graduate Institute of Medical Education and Research; Gursimer Jeet,<br>Thakur, JS; Post Graduate Institute of Medical Education and Research, Department of Community Medicine<br>Prinja, Shankar; Post Graduate Institute of Medical Education and Research, Community Medicine<br>Singh, Meenu; Post Graduate Institute of Medical Education and Research, Advanced Pediatric Centre<br>Paika, Ronika; School of Public Health, Department of Community Medicine, Post Graduate Institute of Medical Education and Research<br>Kunjan, Kunjan; School of Public Health, Department of Community Medicine, Post Graduate Institute of Medical Education and Research<br>Dhadwal, Priya; Post Graduate Institute of Medical Education and Research, School of Public Health
**Primary Subject Heading**:	Evidence based practice
Secondary Subject Heading:	Public health, Evidence based practice, Cardiovascular medicine, Diabetes and endocrinology
Keywords:	Targeted Interventions, NCDs, Risk Factors, Healthy Settings

SCHOLARONE™
Manuscripts

Title Page

Title: Effectiveness of Targeting the Health Promotion Settings for Non-Communicable Disease Control in Developing Countries: Systematic Review Protocol

Authors: Gursimer Jeet[1] MPH, JS Thakur[1] MD, Shankar Prinja[1] MD MSc, Meenu Singh[2] MD, Ronika Paika[1]MPH, Kunjan Kunjan [1]BDS, Priya Dhadwal[1]BDS

Affiliations:
[1]School of Public Health, Post Graduate Institute of Medical Education and Research, Chandigarh, India
[2]Advanced Paediatric Centre, Post Graduate Institute of Medical Education and Research, Chandigarh, India

Type of Manuscript: Systematic Review Protocol

Word count: 289 words abstract, 3774 words main text

Keywords: Targeted Interventions, NCDs, Risk Factors, Healthy Settings

Running Title: Targeted Interventions for Non-Communicable Diseases in Health Promoting Settings

Corresponding author

Dr. Gursimer Jeet
Research Scholar,
School of Public Health,
Post Graduate Institute of Medical Education and Research, Chandigarh
Email Id: gsj_2008@yahoo.com

Contact addresses of all authors:

Dr. Gursimer Jeet
Research Scholar,
School of Public Health,
Post Graduate Institute of Medical Education and Research, Chandigarh
Email Id: gsj_2008@yahoo.com

Dr. JS Thakur
Professor of Community Medicine,
School of Public Health,
Post Graduate Institute of Medical Education and Research, Chandigarh
Email Id: jsthakur64@gmail.com

Dr. Shankar Prinja
Associate Professor,
School of Public Health,
Post Graduate Institute of Medical Education and Research, Chandigarh
Email Id: shankarprinja@gmail.com

For peer review only

For peer review only - http://bmjopen.bmj.com/site/about/guidelines.xhtml

Dr. Meenu Singh
Professor,
Advanced Pediatric Centre,
Post Graduate Institute of Medical Education and Research, Chandigarh
Email Id: meenusingh4@yahoo.com

Dr. Ronika Paika
Research Scholar,
School of Public Health,
Post Graduate Institute of Medical Education and Research, Chandigarh
Email Id: ronikapaika28@gmail.com

Dr. Kunjan Kunjan
MPH Scholar,
School of Public Health,
Post Graduate Institute of Medical Education and Research, Chandigarh
Email Id: dr.kunjan2human@gmail.com

Dr. Priya Dhadwal
MPH Scholar,
School of Public Health,
Post Graduate Institute of Medical Education and Research, Chandigarh
Email Id: priyadhadwal.star@gmail.com

For peer review only

ABSTRACT

Title: Effectiveness of Targeting the Health Promotion Settings for Non-Communicable Disease Control in Developing Countries: Systematic Review Protocol

INTRODUCTION:

Settings-based approaches to health promotion, involving holistic and multi-disciplinary methods which integrate action across risk factors are important. Major advantage of focusing on these settings is the continuous as well as intensive contact with the participant. Despite the apparent advantages of addressing Non-Communicable Diseases (NCDs) using targeted interventions for several developed country settings, a relative lack of evidence of effectiveness of such interventions in developing countries has led to poor allocation of resources towards these interventions. The focus is therefore on the settings rather than any one condition and we therefore expect the findings to generalise to NCD prevention and control efforts. We intend to estimate the effectiveness of targeted interventions in developing countries.

METHODS AND ANALYSIS:

We will search PubMed, EMBASE, OVID, WHO Library and The Cochrane Library from the year 2000 to September, 2016 without language restrictions. Study designs to be included will be Randomised Controlled Trials. The primary outcome of effectiveness will be the percentage change in population having different behavioural risk factors. Subgroup analyses will be performed and sensitivity analyses will be conducted to assess the robustness of the findings.

ETHICS AND DISSEMINATION:

No ethical issues are foreseen. The Institute Ethics Committee of the Post Graduate Institute of Medical Education and Research approved the doctoral research protocol under which this review is being done. Dissemination will be done by submitting scientific articles to academic peer-reviewed journals. We will present the results at relevant conferences and meetings.

STUDY DESIGN: Systematic Review

REGISTRATION NUMBER: PROSPERO 2016:CRD42016042647

For peer review only

For peer review only - http://bmjopen.bmj.com/site/about/guidelines.xhtml

For peer review only

ARTICLE SUMMARY

Strengths and limitations of the study

1. The review will contribute towards evidence synthesis on effectiveness of settings-based approach for reducing burden of NCD risk factors in resource constrained settings

2. This will be first of its kind review resulting in synthesis of evidence from Randomised Controlled Trials on targeted settings in developing countries

3. The synthesis of evidence can probably act as an advocacy tool for better resource allocation towards settings-based approach

4. Due to resource constraints, focus is limited to developing countries. A more comprehensive review would have been, synthesis of evidence available globally or comparison of the effects of the design in developed and developing settings

REVIEW PROTOCOL

Effectiveness of Targeting the Health Promotion Settings for Non-Communicable Disease Control in Developing Countries: Systematic Review Protocol

Background

Prevalence of risk factors of non-communicable diseases (NCDs) among populations in developing countries is high.[1,2] In 2012, NCDs contributed towards 68% of total deaths in the world with 80% of deaths in developing countries.[2] The figure is more alarming than it appears as 52% of the NCD deaths that occurred were premature, i.e. among people aged 30-70 years.[2] In the year 2012, 48% of all deaths in South-east Asia were premature.[3] As the 4 major NCDs i.e., Cardiovascular Diseases, Diabetes, Cancer and Stroke share 4 modifiable risk factors (Tobacco, Alcohol, Diet and Physical activity), the prevention component is very crucial for this group of diseases. There is a global call to develop interventions that address the risk factors using different health promotion approaches.[4,5]

There is good evidence that health promotion interventions focusing on changing lifestyle behaviors are more effective if conducted by targeting settings[6] rather than population based approaches alone. Therefore, settings-based approaches to health promotion, involving holistic and multi-disciplinary methods which integrate action across risk factors have become important.[4,7] Major advantage of focusing on these settings is the continuous as well as intensive contact with the participant. It is also indicated that support from staff, suitable infrastructure, physical environment and health promoting policies have potential to positively influence the health of a person especially in these kinds of settings.[8] In order to decrease the burden of proximal and distal determinants of NCDs which has increased due to globalisation and urbanisation, settings-based approaches may be implemented in a complete and inclusive manner by targeting singular or multiple settings simultaneously(Figure 1). These have to be guided by principles of equity, supportive environments, empowerment, community participation and multi-sectoral partnerships with strengthened health services to achieve maximum benefit in terms of disease prevention.[9] The framework given as Figure 1 helps to understand how focusing on risk factors prevalent in the “settings” can help in finding a solution to NCDs at a larger scale. Efforts can be channelled through general population or sub-populations. Additive or multiplicative effect of working through settings can be expected, as some settings are very closely related like workplaces and homes, schools and homes. A change in behavior patterns and in turn risk profile with respect to non-communicable diseases can be expected to percolate from one setting to the other. This approach is likely to alter behavior patterns in the form of decreased risk factor levels and consequently a shift in the incidence and prevalence of cardiovascular diseases, diabetes, cancers and chronic respiratory diseases. Finally, the rate of increase in burden of morbidity and mortality due to non-communicable diseases can be slowed down by using targeted interventions in various settings.

In this review, strategy of targeted interventions is being evaluated for control of NCD risk factors under different health promoting settings mentioned in the framework. The settings approach has proven to be effective in control of several other risk factors or diseases such as HIV[10-12], immunization,[13] etc. Despite the apparent advantages of addressing NCDs using targeted interventions for several developed settings[14,17], a relative lack of evidence of effectiveness of such interventions in developing countries[18]

For peer review only - http://bmjopen.bmj.com/site/about/guidelines.xhtml

has led to poor allocation of resources towards these interventions. The systematic review aims to assess existing evidence on effectiveness to guide health promoting sites to accept, adopt, and correctly deliver targeted interventions for NCDs.

Methods

In reporting the protocol for this review, PRISMA-P checklist has been adhered to and is provided as a Supplementary File along with the main manuscript (Supplementary File 1).[19]

Population: The populations of interest will be the different stakeholders involved in design, implementation and sustainability of targeted NCD control interventions in different settings from developing countries.

Intervention details: Interventions here refer to use of "settings-based approach for delivery of health promotion interventions." Settings here are defined as, "The place or social context in which people engage in daily activities in which environmental, organisational, and personal factors interact to affect health and wellbeing."[20] They can be identified as having physical boundaries, a range of people with defined roles, and an organizational structure. Schools and workplaces have been popular since long as a setting for implementation of such interventions.[6] However, the definition and scope of settings has widened since last few decades with acceptance of a wide range of health promoting settings, such as cities, villages, municipalities/communities, workplaces, markets, home, hospitals, islands, prisons and old age homes.[9]

For school based targeted interventions studies in primary, middle, high secondary, and central schools (a selective secondary education school in some countries or the schools that are affiliated to central government in some countries) irrespective of their being private, public or aided (school receiving a part of its maintenance costs from public funds but retaining control over appointments and religious instruction) will be considered for inclusion. The inclusion criterion for mean age of students will be age between 5-18 years. For college, the trials recruiting students from institutions with bachelor, master's courses or other professional recognized courses will be included in the study. For schools and colleges, participants can be teaching staff and non-teaching staff, in which case trials may be classified as workplace settings. Participants may be individuals, classes, groups or whole institution.

For work site interventions, we will include trials set in government, non-government, aided institutions, organised or unorganised sectors set in developing countries. These may be involved in implementation of NCD control interventions or programs by the means of encouragement, accreditation or enforcement. Participants may be individuals, group of workers or whole institute.

Comparator: Routine care or enhanced routine care

Inclusion and Exclusion Criterion: Since we are focusing upon the settings, cluster-RCTs will be the most eligible study design for inclusion. However individual RCTs will be included if followed in any study. The controlled trials, quasi-experimental study designs may be the feasible design for intervention implementation, but in light of their inherent weaknesses such as lack of control area or lack of random assignment; internal validity gets reduced and causal inference becomes difficult.[21] Therefore, these study designs will be excluded from the review.

Only those interventions are selected that are disseminated using settings-based approach. We will include studies comparing healthy settings-based strategies with target of promoting behaviours that are healthier when it comes to undertaking physical activity, patterns with respect to diet, use of tobacco,

For peer review only - http://bmjopen.bmj.com/site/about/guidelines.xhtml

consumption of alcohol against usual practice or enhanced usual care. A protocol detailing methodology of this review was registered at PROSPERO[22]. Strategies of intervention may include multidisciplinary expert training groups, education and training part of strategy may involve discussions, group tasks, regular tests, etc., followed by expert group recommendations for quality improvement, reminder letters and follow up assessments for overall reduction in risk factors.[23] The interventions under scope of this process can be singular or with multiple components. These can be directed at particularly individuals, groups or complete institutions.[23,24] The model of delivery of interventions can belong to (but is not limited to) change in organisation policies or practices, enhanced family support, social networking, change in physical environment or routine practices.

Types of outcome measures: Selection of outcomes for the review is guided by nature of interventions under study which focus primarily on promoting the healthy behaviours. We have selected change in participant behaviour and a consequent shift in their physical (e.g., weight, blood pressure), biochemical or cognitive parameters as primary outcomes as these are directly related to participant's health status. Additionally, as secondary outcomes, we will report rate of use of services or cost/cost effectiveness of implementing interventions.

Primary outcomes

1. Self- reported change in Behavioural Parameters
2. Changes in Physiological or Clinical parameters
3. Changes in knowledge and attitudes towards NCD Risk Factors

Secondary outcomes (outcomes reported along with health outcomes)

1. Change in utilisation of health promotion related services (e.g., smoking quit-lines and counselling)
2. Costs/Cost Effectiveness of the interventions

Search methods for identifying studies: Due to the diversity of population, settings in which interventions may be implemented and outcome types can be observed, a multi-stage electronic search strategy has been developed to identify relevant publications. A draft search strategy has been submitted as Supplementary File 2. Searches of published literature on effectiveness of NCD control interventions delivered through targeted health promoting settings (focused on developing countries) will be done in the following biomedical, and general reference electronic databases, without restriction to language, however the publication year is restricted to 2000-2016: PubMed (2000–2016); Excerpta Medica Database (EMBASE) (2000–2016); OVID and World Health Organization (WHO) library. Cochrane data base of Systematic Reviews is another source of information that is proposed to be reviewed for relevant literature. The time period has been chosen considering the evidence that NCD prevention and control priorities have been set recently in developing countries primarily after World Health Organization's call for global action. World Bank list (2016) will be used to define the countries or regions whose data will be used for effectiveness estimation.[25]

Further related studies will be identified by examining the reference lists of articles which have been already identified through above mentioned database searches. In similar way, bibliographies of

For peer review only - http://bmjopen.bmj.com/site/about/guidelines.xhtml

systematic and non-systematic review articles will also be examined to identify further related studies. Authors or trial investigators, for further information, will be contacted if any query pertaining to methodology, study outcome etc., will arise. Abstracts and full text of identified manuscripts will be reviewed. Reference lists of systematic reviews obtained in the initial scoping reviews will be screened for relevant trials. Studies examining effectiveness and those evaluating cost-effectiveness will be reviewed separately.

Searching other sources: The conference proceedings will be searched by hand wherever feasible (pending availability) for the last 5 years for conferences held in the field of NCDs, health promotion and health systems. In addition, trial registration Website and WHO International Clinical Trials Search Portal[26] will be searched to identify trials in process or to compare the published trials with registered protocols. In addition trial registration websites of individual developing countries such as Chinese Clinical Trials Registry[27], Clinical Trials Registry (India)[28], Clinical Research Information Service (Republic of Korea)[29], Pan African Clinical Trial Registry[30], Sri Lanka Clinical Trials Registry[31], Brazil[32] will be explored. We will also search different relevant web sources like Grey Literature Report (www.greylit.org) and Eldis (www.eldis.org) for relevant grey literature.

Selection of trials and data abstraction: Two researchers (GJ/KK/PD/RP) will screen the titles and abstracts individually and in a manner independent of each other to identify their relevancy. Titles and the abstracts of articles identified using searches will be imported to EndNote.[33] All of the duplicates discovered in the process will be removed. In addition to assessment of every abstract and title by both researchers, the supervisor (JS) will further independently assess 50% of the abstracts. Retrieval of full text articles will be done for articles for which the selection criteria is met or declared unclear by both reviewers. If both reviewers agree to the situation of even one selection criteria not being met then full text retrieval will not be done. For the titles/abstracts where all selection criteria found categorised as 'no' by one reviewer were categorized entirely opposite or unclear by other reviewer, the final decision will be to retrieve the full text of the article for better review of selection criteria and if any disagreements are found during both stages then they will be resolved by discussion with third reviewer (JS). Studies with a control group other than routine care, studies with a sample size <30 and studies without a clearly defined intervention will be excluded. Articles written in a language other than English will be translated whenever possible. Multiple publications of the same study will be identified, grouped together and represented by a single reference.

Data extraction and management: Data will be extracted using data abstraction form which will be developed on Microsoft Office Excel 2010. The data abstraction tool will be piloted on a random sample of 5 trials and modified as per the feedback from team. Data will be extracted by one reviewer (GJ) and checked against the original paper by a second independent reviewer (RP/KK/PD). Any disagreements during the process will be subject to discussion to reach a solution and if not resolved by discussion between the two then the issue will be resolved as per final decision of the third reviewer (JS). The data extraction for effectiveness assessment will be carried out in following domains: Study description (authors, aims, objectives, ethical issues); Trial details (details of location, population, intervention and control used); Intervention details (allocation methods, theoretical basis,

For peer review only - http://bmjopen.bmj.com/site/about/guidelines.xhtml

content of intervention, delivery, duration, targeted setting); Outcome measures used and results with sufficient information; targeted risk factors, type of settings-based health promotion.

Quality as well as risk of bias assessment in included studies

Risk of bias assessment will be done by using 'Cochrane risk of bias tool'. [34] Two authors will undertake assessment of risk of bias (GJ/PD/KK) and arbitration will be done by a third author in case of disagreements. (JS/MS/SP)

In order to assess the quality of a trial, Effective Public Health Practice Project Quality Assessment Tool (EPHPP) risk of bias tool will be used in addition to 'Cochrane risk of bias tool'.[35] Inter-rater reliability coefficients for exclusion as well as quality rating of the reviews will be reported.[36] The interpretation of findings will contain risk of bias assessment as risk of bias table. No restrictions will be made in the analysis on the basis of degree of risk of bias. For each primary outcome, a separate presentation of the risk of bias will be documented with respect to blinding and incomplete outcome assessment. Overall strength of evidence assessed using EPHPP tool will be presented by inclusion in 'Characteristics of included studies' table.

Data Analysis

Where data will allow, we will undertake meta-analysis. We will report the mean differences between change in intervention and control group with respect to quantitative data outcomes. In case measurement of same quantitative data outcomes has been undertaken in dissimilar manner, then we will report mean differences (SMD) between the intervention and control group. If we encounter a situation where we don't find a change per group, we will make use of end-values where randomization was successful. We will not include studies in meta-analysis in cases where there is non-availability of information on change per group and if there is reasonable risk of selection bias. For dichotomous outcomes, relative risk (RR) will be reported. 95% confidence intervals (CIs) will be reported with all of the effect estimates.

If it is unclear whether given study is a Cluster RCT or not, the original investigator of the study will be contacted for further information. In case a situation is encountered where the original investigators have not included the clustering effect in their analyses, we will use standard methods as suggested by Cochrane group. First we will request for provision of data for individual participants, so that an intra-cluster correlation coefficient (ICC) can be calculated, and the data can be re-analysed. In case we can't get primary data, we will try to search the literature for appropriate ICC and make adjustments in the sample size in accordance with it. Correct effect estimates and standard errors from cRCTs (Cluster randomised controlled trials) will be pooled using generic inverse-variance method. [37] Results will be marked as re-analysed if the data is re-analysed. If re-analysis would not be possible, it will be mentioned clearly.

For peer review only - http://bmjopen.bmj.com/site/about/guidelines.xhtml

In cases where measurement of outcomes was done in participants at more than one point of time, grouping of outcomes will be undertaken for similar time points. The minimum duration for which trials must have been implemented for extraction of outcomes is 3 months.

For the interventions with multiple comparison groups, all groups that meet the inclusion criteria will be included in the review. In trials with more than two comparisons, the relevant experimental and control groups will be combined to make a single pair wise comparison. If this combining of the groups is not possible, we will make multiple pair wise comparisons by dividing the sample size of the shared intervention group evenly across the comparison groups to avoid double counting of participants.

In cases where we find that data is lacking in clarity or there are missed values with respect to study methodology, participants lost to follow-up, outcome data, or statistical techniques primary author followed by corresponding author of the study will be contacted through email. We will keep a condition of three reminders for requesting missing data from the authors. In case we do not obtain a response after these, outcome will not be included for the purpose of analysis. All of the data for missing outcomes will be reported in data extraction form and in the risk of bias table.

Assessment of reporting biases

If there are more than ten trials for an outcome, the likelihood of reporting bias will be explored by using funnel plots. A visual assessment of the funnel plots will be done for sources of asymmetry such as small study effects, publication bias, or others. If small-study effects are found to result in asymmetry then further sensitivity analysis will be undertaken to show its effects on the pooled results.

Data synthesis

Detailed narrative synthesis of the results will be done for different settings and risk factors addressed. We will undertake separate meta-analyses with respect to each outcome. In order to incorporate any existing heterogeneity, the random-effects model will be utilized. A forest plot will be formulated for each comparison. However, the results of different outcomes under the trials will be pooled only if we find minimum of two studies and the studies are sufficiently homogeneous (I^2statistic $< 75\%$). If either of the above mentioned two criterion is not met, synthesis of results will be done only in a narrative manner. We will undertake Meta-analysis using RevMan. STATA software will also be used for analyzing the data for publication bias because the publication bias tests such as Eggers test are not applicable in RevMan.[38] For the primary outcomes of this review, a summary of findings table will be incorporated to take into account the number of participants and studies for each outcome, a summary of the intervention effect and a measure of the quality of evidence for each outcome according to GRADE considerations.[39]

Subgroup analysis and investigation of heterogeneity

Outcomes of interventions identified under the review will be grouped by risk factors/diseases and type

For peer review only - http://bmjopen.bmj.com/site/about/guidelines.xhtml

of setting. Such types of analyses will permit exploration of clinical as well as methodological heterogeneity between studies.[40] In order to make a comparison between different subgroups, we will conduct a standard heterogeneity test in RevMan 2012. This will be done by determining the I^2 statistic. Assessment of heterogeneity, or the variability among the studies included in a meta-analysis will be done using visual inspection of overlap of confidence intervals as well as by assessing statistical heterogeneity with the Chi squared test. For I^2 statistic above 75% (an indication of substantial heterogeneity) results will not be pooled. We plan to undertake sensitivity analysis to make an assessment of how study size, study quality affect the review findings.

Ethics and dissemination:

No ethical issues are foreseen. The Institute Ethics Committee of the Post Graduate Institute of Medical Education and Research approved the doctoral research protocol under which this review is being done. Dissemination will be done by submitting scientific articles to academic peer-reviewed journals. We will present the results at relevant conferences and meetings.

Discussion:

Sustained support in international health promotion research has led to generation of vast volume of literature on settings-based approach. Through this review we aim to explore their success in NCD prevention. This will help to conclude and generalize the targeted interventions for NCDs in different settings among developing countries, thus, ultimately guiding the resource allocation towards preventive and curative services using settings-based approach.

For peer review only

For peer review only - http://bmjopen.bmj.com/site/about/guidelines.xhtml

Supplementary File 1: PRISMA-P Check list

Supplementary File 2: Search Strategy

Contributorship Statement:

1. Concept and design of study: JS Thakur, Gursimer Jeet, Shankar Prinja, Meenu Singh
2. Refining the methodology and data abstraction tools: Gursimer Jeet, Kunjan Kunjan, Priya Dhadwal, Ronika Paika
3. Drafting the manuscript: Gursimer Jeet, Kunjan Kunjan, Priya Dhadwal, Ronika Paika
4. Revising manuscript critically for important intellectual content: Meenu Singh, Shankar Prinja, JS Thakur
5. Final approval of the version to be published: JS Thakur, Shankar Prinja, Meenu Singh

Competing Interests: There are no competing interests pertaining to the review.

Funding: This research is not receiving any specific grant from any funding agency in the public, commercial or not-for-profit sectors.

Data Sharing Statement: Detailed search strategy, technical appendices, statistical codes and dataset will be provided along with review and will be shared at the time of review publication.

For peer review only

References

1. Thakur JS, Jeet G. Burden of Non Communicable Diseases: Mortality, Morbidity and Trends. Public Health Approaches to Non-Communicable Diseases. New Delhi: Wolters Kluwer 2015.
2. WHO. Global Status Report on noncommunicable diseases. Geneva, Switzerland: World Health Organisation, 2014:273.
3. WHO SEARO. Noncommunicable Diseases [Internet]. New Delhi: WHO SEARO; 2016[cited 2016 September 26]. Available from: http://www.searo.who.int/entity/noncommunicable_diseases/en/28/01/2017.
4. Bovet P, Paccaud. F. Cardiovascular disease and the changing face of global public health: a focus on low and middle income countries. *Public Health Reviews* 2012;33(2):397-415.
5. World Health Organization. NCD Global Monitoring Framework. [Internet]. Geneva: World Health Organisa-tion; 2013 [28/01/2017]. Available from: http://www.who.int/nmh/global_monitoring_framework/en/27/01/2017.
6. Verstraeten R, Roberfroid D, Lachat C, Leroy JL, Holdsworth M, Maes L, et al. Effectiveness of preventive scho-ol based obesity interventions in low- and middle-income countries: a systematic review. *The American journal of clinical nutrition* 2012;96(2):415-38. doi: 10.3945/ajcn.112.035378 [published Online First: 2012/07/05]
7. Kokko S, Green LW, Kannas L. A review of settings-based health promotion with applications to sports clubs. *Health Promotion International* 2014;29(3):494-509. doi: 10.1093/heapro/dat046 [published Online First: 2013/07/03]
8. Mulligan JA, Walker D, Fox-Rushby J. Economic evaluations of non-communicable disease interventions in developing countries: a critical review of the evidence base. *Cost Effectiveness and Resource Allocation: C/E* 2006;4:7. doi: 10.1186/1478-7547-4-7 [published Online First: 2006/04/06]
9. WHO. Healthy Settings [Internet]. Geneva 2016 [Available from: http://www.who.int/healthy settings/en/ accessed 23/09/2016.
10. Clatts MC, Goldsamt LA, Giang LM, et al. Sexually transmissible infection and HIV prevention and treatment for young male sex workers in Vietnam: findings from the SHEATH intervention. *Sexual Health* 2016 doi: 10.1071/sh16051 [published Online First: 2016/09/09]
11. Wang PC, Mwango A, Moberley S, Brockman BJ, Connor AL, Kalesha-Masumbu P, et al. A Cluster Randomised Trial on the Impact of Integrating Early Infant HIV Diagnosis with the Expanded Programme on Immunization and HIV Testing Rates in Rural Health Facilities in Southern Zambia. *PloS One* 2015;10(10):e0141455. doi: 10.1371/journal.pone.0141455 [published Online First: 2015/10/30]
12. Prinja S, Bahuguna P, Rudra S, Gupta I, Kaur M, Mehendale SM et al. Cost effectiveness of targeted HIV prevention interventions for female sex workers in India. *Sexually Transmitted Infections* 2011;87(4):354-61. doi: 10.1136/sti.2010.047829 [published Online First: 2011/03/31]
13. Pati S, Ladowski KL, Wong AT, Huang J, Yang J. An enriched medical home intervention using community health workers improves adherence to immunization schedules. *Vaccine* 2015;33(46):6257-63. doi: 10.1016/j.vaccine.2015.09.070 [published Online First: 2015/10/06]
14. Evans CE, Albar SA, Vargas-Garcia EJ, Xu F. School-Based Interventions to Reduce Obesity Risk in Children in High- and Middle-Income Countries. *Advances in Food and Nutrition Research* 2015;76:29-77. doi: 10.1016/bs.afnr.2015.07.003 [published Online First: 2015/11/26]
15. Jago R, Rawlins E, Kipping RR, Wells S, Chittleborough C, Peters TJ, et al. Lessons learned from the AFLY5 RCT process evaluation: implications for the design of physical activity and nutrition interventions in schools. *BMC Public Health* 2015;15:946. doi: 10.1186/s12889-015-2293-1[published Online First: 2015/09/25]
16. Jones RA, Lubans DR, Morgan PJ, Okely AD, Parletta N, Wolfenden L, et al. School-based obesity prevention interventions: Practicalities and Considerations. *Obesity Research & Clinical Practice* 2014;8(5):e497-510. doi: 10.1016/j.orcp.2013.10.004 [published Online First: 2014/09/30]
17. Skouteris H, Hill B, McCabe M, Swinburn B, Busija L. A parent-based intervention to promote healthy eating and active behaviours in pre-school children: evaluation of the MEND 2-4randomized controlled trial. *Pediatric Obesity* 2016;11(1):4-10. doi: 10.1111/ijpo.12011 [published Online First: 2015/02/28]

For peer review only

For peer review only - http://bmjopen.bmj.com/site/about/guidelines.xhtml

18. Frieden TR. A framework for public health action: the health impact pyramid. *American Journal of Public Health* 2010;100(4):590-5. doi: 10.2105/ajph.2009.185652 [published Online First: 2010/02/20]
19. Shamseer L, Moher D, Clarke M, Ghersi D, Liberati A, Petticrew M, et al. Preferred reporting items for 450 systematic review and meta-analysis protocols (PRISMA-P) 2015: elaboration and explanation. *BMJ (Clinical research ed)* 2015;349:g7647. doi: 10.1136/bmj.g7647 [published Online First: 2015/01/04]
20. World Health Organisation. The WHO Health Promotion Glossary. Switzerland: WHO, 1998.
21. Harris AD, McGregor JC, Perencevich EN, Furuno JP, Zhu J, Peterson DE,et al. The Use and Interpretation of Quasi- Experimental Studies in Medical Informatics. *Journal of the American Medical Informatics Association: JAMIA* 2006;13(1):16-23. doi: 10.1197/jamia.M1749
22. Jeet G, Thakur JS, Prinja S, Singh M, Paika R. Effectiveness of targeted interventions for prevention and control of noncommunicable diseases in academic, worksite and faith based settings of developing countries. *PROSPERO 2016:CRD42016042647* 2016
23. Mattila R, Malmivaara A, Kastarinen M, Kivelä SL, Nissinen A. Effectiveness of multidisciplinary lifestyle intervention for hypertension: a randomised controlled trial. *Journal of Human Hypertension* 2003; 17:199-205.
24. Prabhakaran D, Jeemon P, Goenka S, Lakshmy R, Thankappan KR, Ahmed F, et al. Impact of a Worksite Program on Cardiovascular Risk Factors: A Demonstration Project in an Indian Industrial Population. *JACC* 2009;53(18):1718-28.
25. The World Bank. World Bank Country and Lending Groups [Internet]. Washington, DC: World Bank; 2016 [Available from: https://datahelpdesk.worldbank.org/knowledgebase/articles/906519-world-bank-country-and-lending-groups27/01/2017.
26. WHO. International Clinical Trials Registry Platform (ICTRP). Geneva, Switzerland.
27. Chinese Clinical Trial Register (ChiCTR). West China Hospital, Sichuan University, China: Chinese Evidence-Based Medicine Center, 2005-2015.
28. DST, WHO, ICMR. Clinical Trials Registry-India (CTRI). New Delhi: National Institute of Medical Statistics, ICMR, 2016.
29. Clinical Research Information Service (CRIS). Republic of Korea: Korea Centers for Disease Control and Prevention (KCDC), 2016.
30. SACC, EDCTP. Pan African Clinical Trials Registry (PACTR). South Africa: South African Cochrane Centre, South African Medical Research Council, 2016.
31. Sri Lanka Clinical Trials Registry. Colombo, Sri Lanka: Sri Lanka Medical Association.
32. Brazilian Ministry of Health, PAHO, FIOCRUZ. The Brazilian Clinical Trials Registry (ReBEC). Rio de Janeiro, RJ-Brazil.
33. Thomson Reuters. EndNote X6 [program]. New York, USA: Thomson Reuters, 2012.
34. Higgins JPT, Altman DG, Gøtzsche PC, Jüni P, Moher D, Oxman AD, et al. The Cochrane Collaboration's tool for assessing risk of bias in randomised trials. *BMJ (Clinical research ed)* 2011;343 doi:10.1136/bmj.d5928
35. Armijo-Olivo S, Stiles CR, Hagen NA, Biondo PD, Cummings GG. Assessment of study quality for Public systematic reviews: a comparison of the Cochrane Collaboration Risk of Bias Tool and the Effective Health Practice Project Quality Assessment Tool: methodological research. *Journal of Evaluation in Clinical Practice* 2012;18(1):12-8. doi: 10.1111/j.1365-2753.2010.01516.x [published Online First: 2010/08/12]
36. McHugh ML. Interrater reliability: the kappa statistic. *Biochemia Medica* 2012;22(3):276-82. [published Online First: 2012/10/25]
37. Centre for Reviews and Dissemination. Core principles and methods for conducting a systematic review of health interventions Systematic Reviews: CRD's guidance for undertaking reviews in health care: CRD, University of York 2008.
38. StataCorp. STATA Statistical Software: Release 13 [program]. College Station, TX: STATA Corp, 2013.
39. Balshem H, Helfand M, HJ Schünemann. GRADE guidelines: Rating the quality of evidence. *J Clin Epidemiol* 2011;64:401-6.
40. Higgins JPT, Green S. Cochrane Handbook for Systematic Reviews of Interventions. Version 5.1.0 [updated March 2011]: The Cochrane Collaboration, 2011.

For peer review only - http://bmjopen.bmj.com/site/about/guidelines.xhtml

For peer review only

For peer review only - http://bmjopen.bmj.com/site/about/guidelines.xhtml

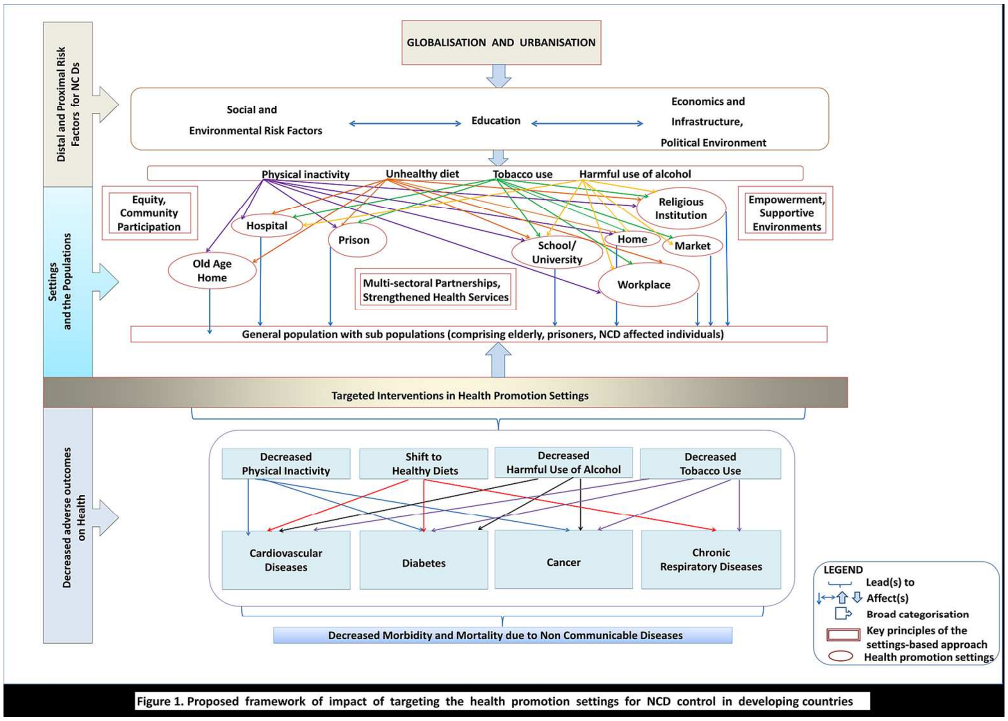

Proposed framework of impact of targeting the health promotion settings for NCD control in developing countries

155x110mm (300 x 300 DPI)

Supplementary File 1: PRISMA-P (Preferred Reporting Items for Systematic review and Meta-Analysis Protocols) 2015 checklist: recommended items to address in a systematic review protocol*

Section and topic	Item No	Checklist item	Reported on Page#
**ADMINISTRATIVE INFORMATION**			
Title:			
Identification	1a	Identify the report as a protocol of a systematic review	P1 (Title)
Update	1b	If the protocol is for an update of a previous systematic review, identify as such	Not an update
Registration	2	If registered, provide the name of the registry (such as PROSPERO) and registration number	P3 (Abstract)
Authors:			
Contact	3a	Provide name, institutional affiliation, e-mail address of all protocol authors; provide physical mailing address of corresponding author	P1, P2
Contributions	3b	Describe contributions of protocol authors and identify the guarantor of the review	P13(Contributorship Statement)
Amendments	4	If the protocol represents an amendment of a previously completed or published protocol, identify as such and list changes; otherwise, state plan for documenting important protocol amendments	NA
Support:			
Sources	5a	Indicate sources of financial or other support for the review	P13 (Funding)
Sponsor	5b	Provide name for the review funder and/or sponsor	NA
Role of sponsor or funder	5c	Describe roles of funder(s), sponsor(s), and/or institution(s), if any, in developing the protocol	NA
**INTRODUCTION**			
Rationale	6	Describe the rationale for the review in the context of what is already known	P6 (Background), Paragraph 1
Objectives	7	Provide an explicit statement of the question(s) the review will address with reference to participants, interventions, comparators, and outcomes (PICO)	P6, P7 (Methods)
**METHODS**			
Eligibility criteria	8	Specify the study characteristics (such as PICO, study design, setting, time frame) and report characteristics (such as years considered, language, publication status) to be used as criteria for eligibility for the review	P7, Last Paragraph
Information sources	9	Describe all intended information sources (such as electronic databases, contact with study authors, trial registers or other grey literature sources) with planned dates of coverage	P8
Search strategy	10	Present draft of search strategy to be used for at least one electronic database, including planned limits, such that it	Supplementary File

For peer review only

		could be repeated	2
Study records:			
Data management	11a	Describe the mechanism(s) that will be used to manage records and data throughout the review	P8 last lines, P9 Paragraph 1 (Data Extraction and Management)
Selection process	11b	State the process that will be used for selecting studies (such as two independent reviewers) through each phase of the review (that is, screening, eligibility and inclusion in meta-analysis)	P9
Data collection process	11c	Describe planned method of extracting data from reports (such as piloting forms, done independently, in duplicate), any processes for obtaining and confirming data from investigators	P8
Data items	12	List and define all variables for which data will be sought (such as PICO items, funding sources), any pre-planned data assumptions and simplifications	P9, Line 268-272
Outcomes and prioritization	13	List and define all outcomes for which data will be sought, including prioritization of main and additional outcomes, with rationale	P7 Line 205,<br>P8 Line 210-212
Risk of bias in individual studies	14	Describe anticipated methods for assessing risk of bias of individual studies, including whether this will be done at the outcome or study level, or both; state how this information will be used in data synthesis	P9, Section: Quality as well as Risk of Bias Assessment
Data synthesis	15a	Describe criteria under which study data will be quantitatively synthesised	P10
	15b	If data are appropriate for quantitative synthesis, describe planned summary measures, methods of handling data and methods of combining data from studies, including any planned exploration of consistency (such as I^2, Kendall's τ)	P11
	15c	Describe any proposed additional analyses (such as sensitivity or subgroup analyses, meta-regression)	P11
	15d	If quantitative synthesis is not appropriate, describe the type of summary planned	P11 Line 327, 336-338
Meta-bias(es)	16	Specify any planned assessment of meta-bias(es) (such as publication bias across studies, selective reporting within studies)	P11, Line 321-325
Confidence in cumulative evidence	17	Describe how the strength of the body of evidence will be assessed (such as GRADE)	P11, Line 338

***NA- Not Applicable**

From: Shamseer L, Moher D, Clarke M, Ghersi D, Liberati A, Petticrew M, Shekelle P, Stewart L, PRISMA-P Group. Preferred reporting items for systematic review and meta-analysis protocols (PRISMA-P) 2015: elaboration and explanation. BMJ. 2015 Jan 2;349(jan02 1):g7647.

For peer review only

Supplemental File 2: **Search Strategy**

Pubmed, Embase, Ovid, Scopus databases will be searched by using specific search terms. The search terms are identified using thesaurus and other controlled vocabulary terms related to health promoting settings along with alternate terms for various diseases under NCDs and their risk factors combined with the name of the developing countries of concern. Alternate terms for developing countries like ‘LMIC’, ‘low income country’, ‘lesser developed countries’, ‘deprived country’ etc will be also be included with country names. Basically the search strategy will include four main sets which will be combined by “AND” Boolean operators. The terms within these main sets will be combined by “OR” Boolean operators.

Titles and abstracts will be searched for “Health Promoting Settings” set. All fields will be searched for “Country” set, “Disease and Risk Factor” set, “Randomised Controlled Trial” set and also for “Intervention” set.

The publication years searched will be 2000-2016.

The terms and sets of search strategy are described in table below:

Set Name	Sub-Set Terms	No. of Terms	Example
Health promoting setting Set	12 sub-sets (School, College, City, Village, Municipality or Community, Workplace, Market, Home, Hospital, Island, Prison, Old Age home)	School- 11<br>College- 23<br>City - 26<br>Village- 10<br>Municipality/ Community- 25<br>Workplace-20<br>Market-12<br>Home- 21<br>Hospital- 24<br>Island- 04<br>Prison- 17<br>Old Age home- 05<br><br>Total= 198	Primary school, govt. school, boarding school, university, educational institute, academy, metropolis, urban area, suburbs, countryside, rural area, society, locality, office, company, firm, plaza, bazaar, household, residence, clinic, nursing home, tertiary care hospital, jail, confinement place, old age home, etc.
Country Set	2 sub-sets (LMIC Country names and Alternate terms for low and middle income countries)	LMIC set - 138<br>Alternate set- 118<br><br>Total= 256	Transitional country, lower income economy, underdeveloped economy, poorer nations, deprived country, etc.
Disease & Risk Factor Set	--	59	NCDs, diabetes, stroke, cancer, CVDs, physical activity, smoking, alcohol, tobacco, diet,

For peer review only

			hypertension, etc.
Intervention Set	--	26	Lifestyle intervention, health behavior intervention, multiple risk factor program, etc.
Randomised Controlled Trial Set	--	16	Cluster randomized, Group randomized, Randomized,etc.

For peer review only
